# Beyond Pain: A Pilot Study of Neurodegenerative and Mitochondrial Pathway Alterations in Sickle Cell Disease Using Platelet Proteomics

**DOI:** 10.3390/medsci14050517

**Published:** 2026-08-26

**Authors:** Keesha Powell-Roach, Ugochi O. Ogu, Erielle Culp, Kalpna Gupta, Eboni I. Lance, Xueyuan Cao, M. Dennis Leo, Daniel Johnson, David Kakhniashvili, Yenisel Cruz-Almeida, Margaret R. Wallace, Diana J. Wilkie, Steven R. Goodman

**Affiliations:** 1 Rutgers Biomedical and Health Sciences, Division of Nursing Science, School of Nursing, Rutgers University, Newark, NJ 07107, USA; 2 Department of Health Promotion and Disease Prevention, College of Nursing, University of Tennessee Health Science Center, Memphis, TN 38103, USAxcao12@uthsc.edu (X.C.); 3 Center for Sickle Cell Disease, University of Tennessee Health Science Center, Memphis, TN 38163, USA; uogu@uthsc.edu (U.O.O.); sgoodma5@uthsc.edu (S.R.G.); 4 Division of Hematology, University of Tennessee Health Science Center, Memphis, TN 38104, USA; 5 School of Pharmacy and Pharmaceutical Sciences, University of California—Irvine, Irvine, CA 92617, USA; 6 Department of Pediatrics, Johns Hopkins University School of Medicine, Baltimore, MD 21287, USA; lance@kennedykrieger.org; 7 Department of Neurodevelopmental Medicine, Kennedy Krieger Institute, Baltimore, MD 21205, USA; 8 Department of Pharmaceutical Sciences, University of Tennessee Health Science Center, Memphis, TN 38163, USA; mleo@uthsc.edu; 9 Office of Research, University of Tennessee Health Science Center, Memphis, TN 38103, USA; djohn166@uthsc.edu (D.J.); dkakhnia@uthsc.edu (D.K.); 10 Community Dentistry, College of Dentistry, University of Florida, Gainesville, FL 32606, USA; 11 Department of Molecular Genetics & Microbiology, College of Medicine, University of Florida, Gainesville, FL 32610, USA; peggyw@mgm.ufl.edu; 12 Biobehavioral Nursing Science, College of Nursing, University of Florida, Gainesville, FL 32611, USA; diwilkie@ufl.edu; 13 Department of Pediatrics, College of Medicine, University of Tennessee Health Science Center, Memphis, TN 38163, USA

**Keywords:** sickle cell disease, platelet proteomics, pain phenotypes, neurodegeneration pathways, mitochondrial dysfunction, oxidative stress

## Abstract

Background: Sickle cell disease (SCD) is a systemic disorder marked by chronic pain and neurocognitive deficits, yet the molecular drivers of these neurocognitive features remain poorly defined. Platelets, central to inflammation and vascular homeostasis, may reflect broad pathophysiologic processes in SCD. Methods: We performed high-resolution mass spectrometry on ultra-purified platelets from 16 adults with SCD and moderate to severe pain (self-reported ≥ 3/10 in the past year), identifying 4196 proteins, of which 1046 were significant (FDR < 0.05). Unsupervised clustering was used to stratify individuals into high- and low-pain phenotypes. Results: Contrary to expectations, canonical pain pathways were not enriched. Instead, significant alterations were observed in neurodegeneration, mitochondrial metabolism, ATP regulation, mitophagy, and tRNA aminoacylation pathways between high- and low-pain phenotypes. High-pain individuals exhibited elevated levels of proteins involved in proteostasis and neurodegenerative disease processes, whereas low-pain individuals showed increased expression of proteins linked to mitochondrial integrity, neuroprotection, and reduced oxidative stress. Protein-protein interaction networks revealed tightly connected clusters within neurodegenerative and central nervous system-related pathways. Disease association analysis ranked neurodegenerative and mitochondrial pathways above traditional hematologic and nociceptive mechanisms. Conclusions: These findings suggest that platelet proteomics may serve as a peripheral window into PNS or CNS vulnerability and cognitive risk in SCD. The enrichment of tRNA aminoacylation and mitochondrial regulation pathways underscores the metabolic complexity of SCD and highlights novel targets for biomarker development and therapeutic intervention.

## 1. Introduction

Sickle cell disease (SCD) is an autosomal recessive hemoglobinopathy resulting from a mutation in the beta hemoglobin (Hb) gene, leading to the production of abnormal hemoglobin with amino acid substitution E6V (HbS for sickle hemoglobin) [1,2,3,4,5] Hemoglobin plays a vital role in oxygen transport, but in individuals with SCD, HbS undergo polymerization under hypoxic conditions, forming rigid, sickled cells [1,2,3,4] These sickled erythrocytes contribute to vaso-occlusion, leading to recurrent episodes of ischemia, tissue hypoxia, and organ damage [1,2,3,4] Over time, repeated vaso-occlusive crises (VOC) result in cumulative injury, manifesting as chronic pain, systemic inflammation, and neurocognitive dysfunction [1,2,3,4,6].

The nervous system, the second-largest system in the body, is highly susceptible to ischemia-reperfusion injury, oxidative stress, and inflammation triggered by VOCs [7,8,9,10,11,12,13,14,15] These insults lead to neuronal damage and dysregulation of pain signaling pathways, exacerbating the burden of chronic pain in SCD [16,17,18] Additionally, sickled erythrocytes contribute to the disruption of neuronal circuits, ultimately leading to peripheral nerve damage, central nervous system damage, and neurocognitive impairment, one of the most frequent neurological complications in SCD [16,18,19,20] All of these types of neural alteration can lead to chronic pain.

Pain in SCD is a complex and multifactorial phenomenon [17,18,21] VOC-induced ischemia-reperfusion injury leads to excessive oxidative stress, activating inflammatory pathways and sensitizing nociceptors [17,18] Additionally, erythrocyte hemolysis exposes free hemoglobin and heme, which further contribute to inflammation-mediated pain [18,22,23] The nervous system, being highly vulnerable to ischemic stress, undergoes structural and functional changes over time, exacerbating the burden of neuropathic and inflammatory pain [18,22,23] The repetitive inflammatory insults inflicted by VOCs lead to peripheral and central sensitization [16,17,18], prolonging pain perception and complicating disease management [24,25].

Although platelets are traditionally recognized for their role in hemostasis, emerging evidence supports their involvement in pain modulation and neurocognitive processes [18,26,27] Proteomics provides a powerful systems-level approach for investigating these mechanisms by enabling large-scale characterization of protein expression and pathway dysregulation [25,28,29,30] Unlike genomic analyses, proteomic profiling captures dynamic downstream cellular responses that more directly reflect physiologic and pathophysiologic states [25,29,31,32] Platelets represent an informative yet underexplored biospecimen in SCD because they are central mediators of thromboinflammation, vascular signaling, oxidative stress, and coagulation, while also sharing several functional and signaling characteristics with neuronal cells [23,26,27,33] However, increasing evidence suggests that platelet proteins may reflect broader central nervous system (CNS) and peripheral nervous system (PNS) processes, including mitochondrial dysfunction, oxidative stress responses, proteostasis, and neurodegenerative signaling [26,33,34].

Recent work in neurodegenerative and chronic inflammatory disorders has identified strong links among mitochondrial dysfunction, impaired oxidative phosphorylation, redox imbalance, disrupted protein quality-control pathways, and chronic pain phenotypes [35,36,37] Proteins involved in mitochondrial maintenance, electron transport, glutathione metabolism, aminoacyl-tRNA synthesis, and ubiquitin-mediated proteostasis have been implicated in neuronal vulnerability and persistent pain signaling [35,36,38,39] However, these pathways remain poorly characterized in SCD. A proteomic approach may therefore provide novel insight into the biological mechanisms underlying pain heterogeneity in SCD and identify molecular signatures associated with high-pain phenotypes [18,21,28,29,31].

In this study, we used high-resolution mass spectrometry to characterize the platelet proteome of adults with SCD and chronic pain. In this proof of principle study, we hypothesized that platelet proteomic profiles would be able to distinguish pain phenotypes. By integrating proteomic pathway, and protein-protein interaction analyses, this study aimed to identify biologically relevant mechanisms that may contribute to chronic pain and reveal potential biomarkers and therapeutic targets for pain in SCD.

## 2. Methods

### 2.1. Design and Setting

We used a cross-sectional design. Participants were recruited from the outpatient sickle cell clinic at Regional One Health (ROH) hospital in Memphis, TN. The Institutional Review Boards at the University of Tennessee Health Science Center and ROH hospital approved the study.

### 2.2. Sample

A convenience sample of 16 participants with SCD and chronic pain was recruited for the study. The inclusion and exclusion criteria are described in Table 1. Participant demographic and personal characteristics are presented in Table 2.

### 2.3. Procedure

During routine appointments at the sickle cell clinic at ROH, eligible individuals with SCD were identified by clinic staff and invited to participate in the study. Those who agreed were scheduled for a dedicated research visit, during which they provided informed consent, completed demographic surveys and standardized questionnaires, and had blood drawn for subsequent proteomic analysis.

### 2.4. Instruments

**Figure 1:**
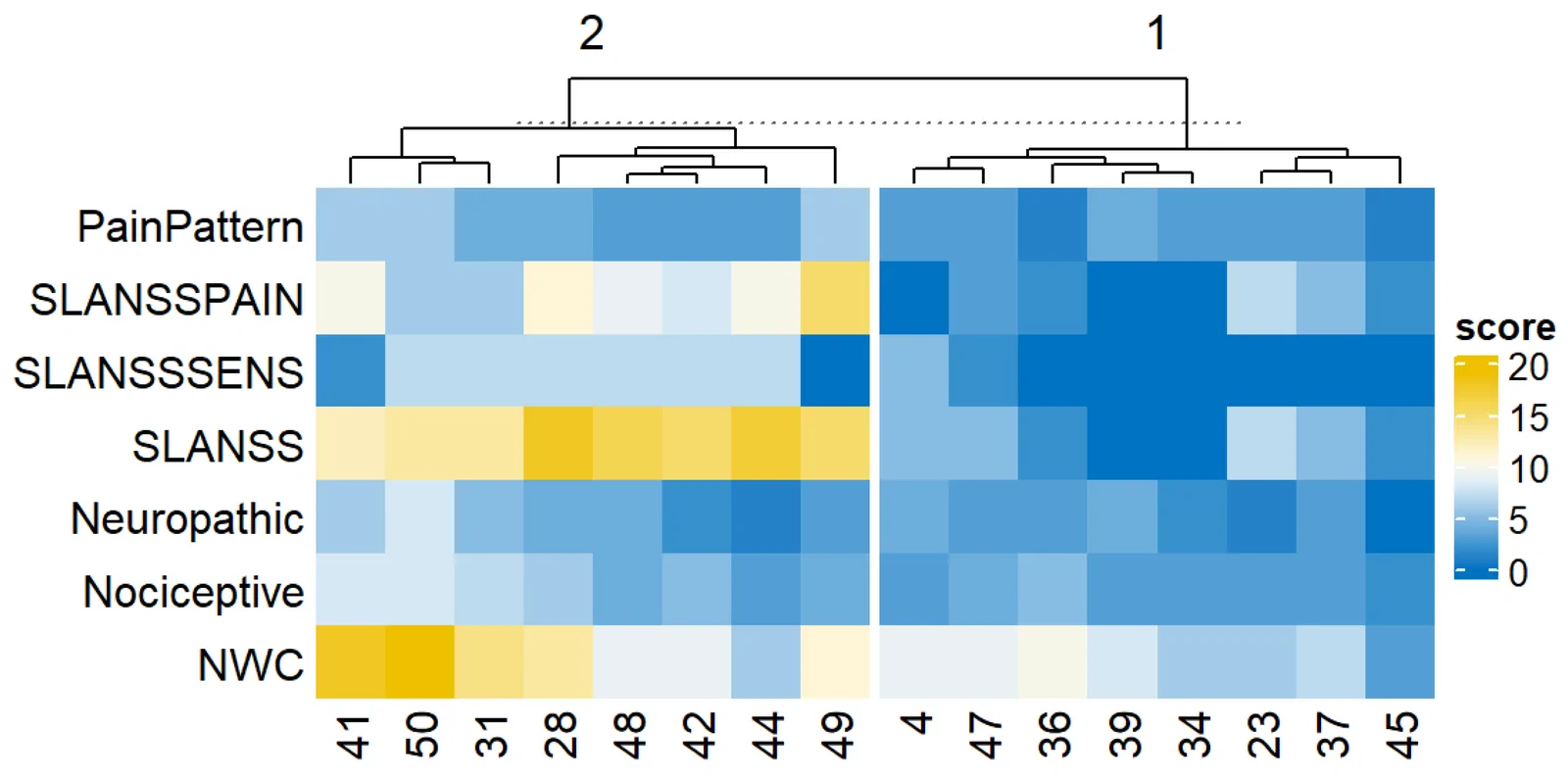
Pain, SLANSS, word counts. The k-means clustering of SLANSS terms led to one high score cluster (**left**) and one low score cluster (**right**). **Legend key**: Pain pattern (0–6), (continuous, intermittent, transient); S-LANSS Pain (self-reported neuropathic symptoms; 0–16); S-LANSS SENS (objective sensory findings; 0–8, with 5 points for allodynia and 3 points for altered pinprick sensitivity); and total S-LANSS score (0–24; ≥12 indicating likely neuropathic pain). MPQ-derived variables include neuropathic descriptors, nociceptive descriptors, and the Number of Words Chosen (NWC), representing the total count of descriptors selected and reflecting pain complexity—color scale ranges from blue to yellow (0 to 20).

PAIN*Report*It^®^ is a web-based adaptation of the McGill Pain Questionnaire (MPQ), widely recognized for its effectiveness in helping patients communicate detailed pain experiences that support accurate diagnosis and treatment planning [40,41] The platform includes MPQ-based screens and captures current pain intensity using a 0–10 Pain Intensity Number Scale (PINS) [42,43] Pain Patterns (0–6) reflect the temporal nature of the participant’s pain experience. Participants self-reported pain at the time of testing: individuals with scores 0–4 were assigned to the low pain group and individuals with 5–10 were assigned to the high pain group. Also used was the Self-report Leeds Assessment of Neuropathic Symptoms and Signs (S-LANSS), a validated, seven-item screening instrument designed to detect neuropathic pain [44,45,46] Scores range from 0 to 24, with values of 12 or higher suggesting the presence of neuropathic pain characteristics [44] In addition to the total S-LANSS score, we used two variables defined in prior publications. SLANSSSENS denotes the sensory-symptom sub-score, computed as the sum of the five symptom-descriptor items (burning, electric-shock–like pain, tingling/pins-and-needles, tactile sensitivity/allodynia, and color/temperature change), yielding a 0–16 range in which higher values reflect greater neuropathic-type sensory burden [44,46,47] SLANSSPAIN is the neuropathic-pain classification flag based on established convention, coded 1 when the total S-LANSS score is ≥12 (indicative of pain with predominantly neuropathic features) and 0 otherwise [44,46,47] Both variables were applied according to previously reported scoring conventions [46,47] Neuropathic descriptors (6–10) were defines as a subset of sensory terms associated with nerve related pain [48] Nociceptive descriptors (20–30) included terms reflecting tissue injury or inflammation [48] The Number of Words Chosen (NWC) represents the total count of pain descriptors selected across all categories and reflects the breadth and complexity of the pain experience, with higher counts indicating more varied or multifaceted pain profiles [49] (Figure 1) No participants selected more than 22 descriptors.

### 2.5. Blood Collection

Peripheral blood samples were obtained from the 16 adult volunteers living with SCD following informed consent. Venipuncture was performed using sterile techniques, and 10 mL of whole blood was collected per individual into vacutainer tubes (ACD—yellow cap). Tubes were gently inverted five times to ensure uniform anticoagulation and processed within one hour of collection.

### 2.6. Preparation of Ultrapure Platelets for Proteomic Analysis

To separate plasma from cellular components, whole blood samples were centrifuged at 200× *g* for 20 min at room temperature without brake [50,51] The platelet-rich plasma (PRP) layer was carefully aspirated to avoid contamination from the buffy coat and erythrocytes [50,51].

The PRP was then centrifuged at 800× *g* for 10 min at 20 °C to pellet platelets. The supernatant was discarded, and the pellet was resuspended in calcium-free phosphate-buffered saline (PBS) [50,51] Washing was repeated twice to minimize plasma protein carryover.

To obtain ultra-pure platelets, we employed a density gradient purification step using OptiPrep™ (Serumwerk, Bernburg, Germany) (iodixanol-based density medium) [50] A discontinuous gradient was prepared by layering solutions of varying iodixanol concentrations (6%, 10%, and 14%) in a sterile ultracentrifuge tube [50] Washed platelet suspensions were gently layered on top of the gradient and centrifuged at 10,000× *g* for 15 min at 20 °C with low acceleration and deceleration without brake [50].

Following centrifugation, the platelet-enriched band was carefully collected from the 10%/14% interface using a sterile pipette [50] This fraction was diluted in PBS and subjected to one additional wash (800× *g*, 10 min at 20 °C) to remove residual OptiPrep and gradient medium [50].

Flow cytometry was used to assess the purity of the isolated platelet preparations and to identify residual leukocyte and erythrocyte contamination. A 10-µL aliquot of each platelet sample was diluted with 90 µL of staining buffer consisting of calcium- and magnesium-free phosphate-buffered saline supplemented with 2% fetal bovine serum (Merck KGaA, Darmstadt, Germany) and 1–5 mM EDTA (Fisher Scientific, Pittsburgh, PA, USA). Samples were incubated with 3.5 µL of human monocyte blocking agent/Fc block (BioLegend, San Diego, CA, USA) for 15 min on ice, followed by staining with fluorochrome-conjugated monoclonal antibodies against CD41-BV605 (BioLegend, San Diego, CA, USA) (5 µL; platelet marker), CD45-Pacific Blue (BioLegend, San Diego, CA, USA) (5 µL; leukocyte marker), CD235a-FITC (BioLegend, San Diego, CA, USA) (1 µL; erythrocyte marker), CD62P-PE (BioLegend, San Diego, CA, USA) (3 µL; platelet activation marker), and CD42b-APC (BioLegend, San Diego, CA, USA) (5 µL; platelet marker). Samples were incubated for 25 min on ice in the dark, washed with 1 mL of staining buffer by centrifugation at 2500× *g* for 10 min at room temperature, and resuspended in 100 µL of staining buffer. When acquisition was delayed, samples were fixed and stored at 4–8 °C in the dark for no longer than 24 h. Single-color compensation controls were prepared using positive and negative compensation beads, and an unstained control was included.

**Figure 2:**
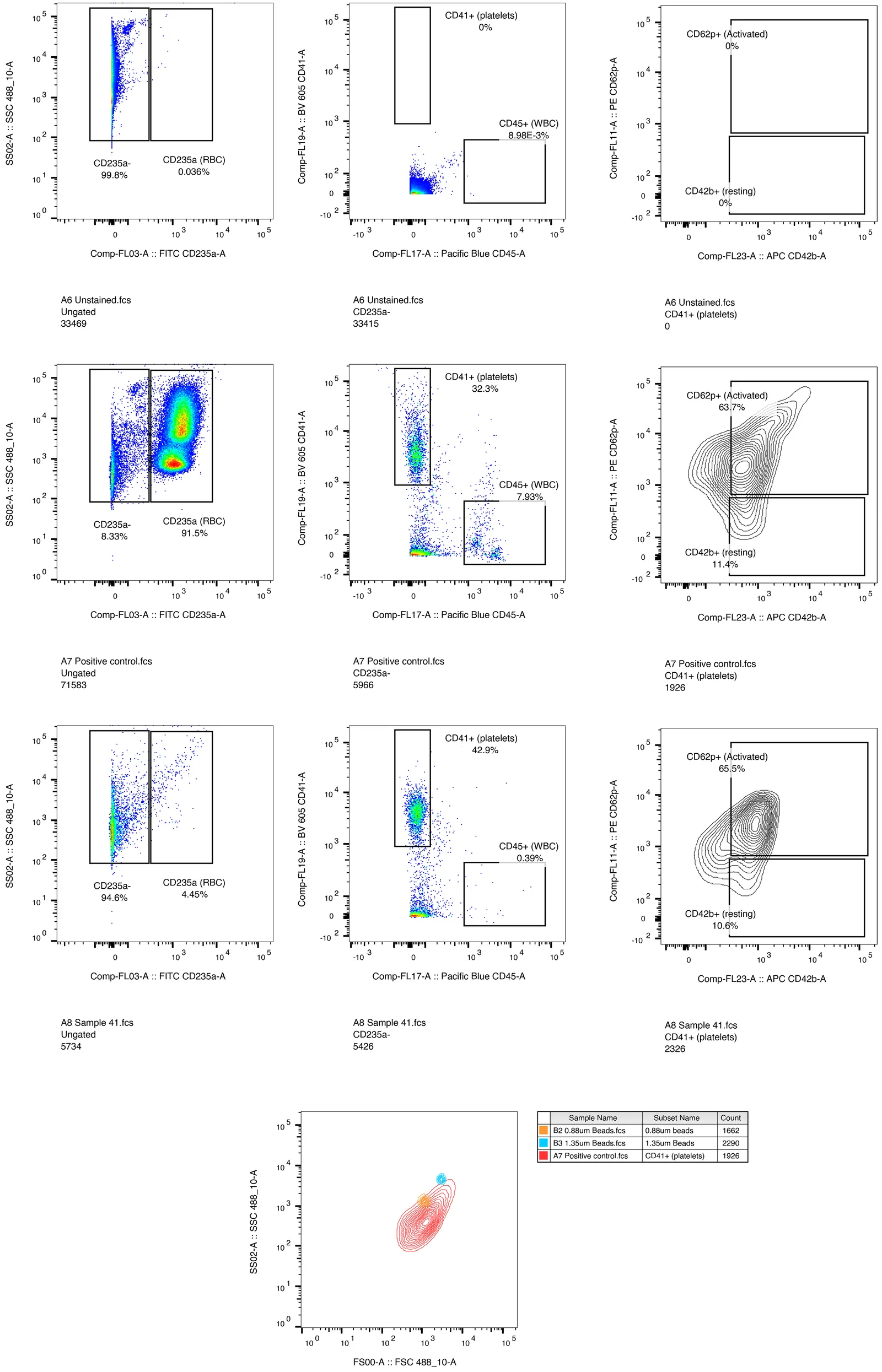
Representative flow-cytometric characterization of isolated platelets. Unstained control, positive control, and representative platelet sample are shown from top to bottom. CD235a identified erythrocytes, CD41 and CD45 distinguished platelets from leukocytes, and CD62P and CD42b characterized platelet activation and identity, respectively. The representative sample demonstrated minimal CD45^+^ leukocyte contamination (0.39%). Data were analyzed using FlowJo v10.8.1.

Samples were acquired and analyzed. After exclusion of debris and cellular aggregates, platelet purity was determined from CD41 and CD45 expression (BioLegend, San Diego, CA, USA), with CD235a (BioLegend, San Diego, CA, USA) used to assess residual erythrocyte contamination. Platelet preparations consistently demonstrated ≥ 98% CD41^+^/CD45^−^ events, confirming effective leukocyte depletion and high platelet specificity. A representative gating strategy and CD41-versus-CD45 dot plot are shown in Figure 2.

### 2.7. Sample Processing for MS Analysis

Each sample containing 25 mg of protein extract in 35 mL of Lysis Buffer was reduced with 10 mM DTT (Fisher Scientific, Pittsburgh, PA, USA) for 45 min at 50 °C in the presence of 100 mM ammonium bicarbonate (Fisher Scientific, Pittsburgh, PA, USA), alkylated with 50 mM iodoacetamide (Fisher Scientific, Pittsburgh, PA, USA) for 20 min at room temperature in the dark with final sample volume of 100 mL, precipitated with 5 volumes of cold acetone (−20 °C, overnight), washed with 50 mL of cold (−20 °C) 90% acetone and air dried for 3 min. The sample proteins were re-dissolved in 50 mL of digestion buffer (100 mM HEPES, pH 8.3) and digested with 1 mg of Pierce Lys-C/Trypsin mixture (Thermo Fisher, A41007—(Fisher Scientific, Pittsburgh, PA, USA)) overnight at 37 °C.

TMTpro-16plex-Labeling: 25 mg of peptides from each subject was labeled using commercial TMTpro-16plex Mass Tag Labeling reagent kit (A44521, Thermo Fisher—(Fisher Scientific, Pittsburgh, PA, USA)) according to the manufacturer’s protocol scaled to 50 mL. Each sample within a set was labeled with one of the 16 different TMT- tags. The labeled set of 16 samples was combined and vacuum dried; the combined dry mixture of labeled peptides was reconstituted in 0.1% TFA at 0.3 mg/mL for further fractionation.

Fractionation: 300 mL (90 mg) of reconstituted labeled peptide mixture was fractionated using Pierce High pH Reversed-Phase Peptide Fractionation kit (Thermo Fisher -(Fisher Scientific, Pittsburgh, PA, USA) according to the manufacturer’s protocol for TMT labeled peptides—8 step fractions (consecutively eluted with 10.0, 12.5, 15.0, 17.5, 20.0, 22.5, 25.0, and 50.0% acetonitrile) were collected. The collected peptide fractions were vacuum dried.

LC-MS: each dried peptide fraction (estimated ~11 mg) was re-dissolved in 65 ul of loading buffer (3% acetonitrile, 0.05% TFA), and 5 mL (estimated ~0.9 mg) was analyzed by LC-MS for peptide/protein identification and quantification. Raw MS data were acquired on an Orbitrap Fusion Lumos mass spectrometer (Thermo Fisher, Waltham, MA, USA) operating in line with Ultimate 3000RSLCnano UHPLC system (Thermo Fisher, Waltham, MA, USA). The peptides were trapped on an Acclaim PepMap 100 nanoViper column (75 µm × 20 mm, Thermo Fisher) at 5 ul/min flow rate and washed with loading buffer for 5 min. The trapped peptides were separated on an Acclaim PepMap RSLC nanoViper column (75 µm × 500 mm, C-18, 2 µm, 100 Å, Thermo Fisher) at 300 nl/min flow rate and 40 °C column temperature using water and acetonitrile with 0.1% formic acid as solvents A and B, respectively. The following multi-point linear gradient was applied: 3%B at 0–4 min, 5%B at 5 min, 23%B at 110 min, 30%B at 120 min, 90%B at 123–133 min, 3%B at 136 –160 min. MS2 acquisition method was used with 3 s cycles and the following MS scan parameters. Full MS scans were performed in the Orbitrap analyzer at 120,000 (FWHM, at *m*/*z* = 200) resolving power to determine the accurate masses (*m*/*z*) of peptides. The data dependent MS analysis was performed on precursor ions with peptide isotopic pattern, charge state 2–6, and intensity of at least 25000. In the MS2 scans (performed for peptide sequence identification), peptide ions were isolated in quadrupole with 0.7 *m*/*z* window, fragmented (HCD, 38% NCE), and the fragment masses and reporter ions were determined in the Orbitrap analyzer at 50,000 (FWHM, at *m*/*z* = 200) resolving power. Dynamic exclusion was applied to data dependent analysis for 45 s.

Post-acquisition analysis of raw MS data: raw MS data acquired for a set of 8 fractions (derived from the same mixture of labeled samples) were grouped for post-acquisition analysis. The analysis was performed within a mass informatics platform Proteome Discoverer 2.4 (Thermo Fisher, Waltham, MA, USA) using Sequest HT search algorithm and human protein database (SwissProt, Homo sapiens, TaxID 9606, v.2022–04–30, 42291 entries). The reversed target database was used as a decoy database. Full tryptic peptides were searched; 2 miss-cleavages were allowed. The searched fixed modifications included: carbamidomethylation of Cys and TMTpro modification of Lys and any N-terminus. The variable modifications included oxidation of Met and acetylation of the protein N-terminus. The precursor, fragment, and reporter ion mass tolerances were set to 10 ppm, 0.02 Da, and 20 ppm, respectively. The raw data were filtered for the precursor ions with S/N of at least 1.5. The PSMs were filtered for further analysis using a delta Cn threshold of 0.05. The q-values were calculated at PSM level (Percolator) and then, at peptide level (Quality algorithm) to control false discovery rate (FDR). The FDR threshold of 0.01 was used to validate and filter the PSMs and the corresponding peptide sequences. The validated/filtered peptides were used for the identification of the candidate precursor proteins. The following criteria were applied to accept (or reject) the identification of a candidate protein or a protein group. The identification of a candidate protein was accepted if at least one of the assigned peptides was unique to that protein. A set of candidate proteins was accepted as an identified protein group if none of the assigned peptides was unique to any protein, but at least one of those peptides was common and unique to that group of proteins. Strict parsimony principle was applied to the protein groups. The ‘best’ protein of a group was displayed as Master protein; the selection was based on the highest parameter with the following priority: the protein score, the number of assigned validated PSMs, the number of assigned validated peptides, and its size (the number of residues). Each candidate precursor protein was scored by summing the PEP values of the assigned peptides. The sum-PEP protein scores were used to calculate the experimental q-values at protein level. The candidate proteins were further validated (without filtering) using 0.01 (strict) and 0.05 (relaxed) FDR thresholds. The reporter ion intensities (signal to noise ratios) were used for quantification of corresponding peptides. The reporter ions quantification values were corrected based on the product data sheet provided with the used TMTpro-16plex Label reagent set; the co-isolation, and average S/N ratio thresholds were set to 50%, and 10, respectively. Unique and razor peptides were used for protein quantification; protein groups were considered for peptide uniqueness. This identified proteins in each platelet sample which were enriched relative to high vs. low pain.

Further processing of the generated data (normalization of the peptide abundances in analyzed samples, calculation of the molar ratios of identical proteins in compared sample groups, and statistical validation of the data with adjustments for multiple testing) was performed by the bioinformatics facility.

### 2.8. Bioinformatics and Statistics

Proteomic dataset underwent standard log2 transformation followed by normalization using cyclic loess methods via the limma package in R version 4.6.1 [52] Descriptive statistics—including mean, median, standard deviation, and coefficient of variation—were computed for each protein. Principal component analysis and Pearson correlation plots were used to assess patterns within the normalized dataset. To identify significant differences between conditions (high vs. low pain), the Wilcoxon rank-sum test was applied. Proteins with *p*-values ≥ 0.05 were excluded from further analysis. The remaining dataset was subjected to Benjamini-Hochberg correction to control for false discovery rate (FDR), and proteins with FDR ≥ 0.05 were also removed. The final list of significantly differentially expressed proteins was visualized using heatmaps in R. Additionally, these targets were analyzed in StringDB to explore associated biological processes and disease relevance [53,54].

Protein identifiers reported in the Results were annotated to HUGO Gene Nomenclature Committee (HGNC)–approved gene symbols and corresponding protein names. Functional summaries and subcellular localization were retrieved from UniProt and cross-checked with HGNC/NCBI Gene, and a brief description was curated for each protein to aid biological interpretation. The resulting annotation table, including gene symbols, protein names, and functional descriptions, is provided in Supplemental Table S1.

The proteomics data generated and analyzed in this study of individuals with SCD will be deposited in the NHLBI BioData Catalyst^®^ (BDC), a secure, cloud-based ecosystem supported by the National Heart, Lung, and Blood Institute. Data will be made available in accordance with NIH and institutional policies governing human subject research, including appropriate de-identification and data use agreements. Metadata and documentation will be provided to facilitate reuse. Where applicable, raw mass spectrometry files will also be deposited in the PRIDE repository and linked to BDC to ensure accessibility for proteomics researchers.

## 3. Results

### 3.1. Unsupervised Clustering and Pain Phenotypes

Unsupervised k-means clustering of pain-related clinical variables, including SLANSS scores, pain descriptors, neuropathic and nociceptive features, and narrative word counts, identified two distinct participant groups corresponding to high- and low-pain phenotypes (Figure 1). Participants within the high-pain cluster demonstrated elevated SLANSS scores, greater neuropathic pain characteristics, and increased pain-related word/word group counts compared to the low-pain cluster.

### 3.2. Proteomic Profiling and Differential Expression

**Figure 3:**
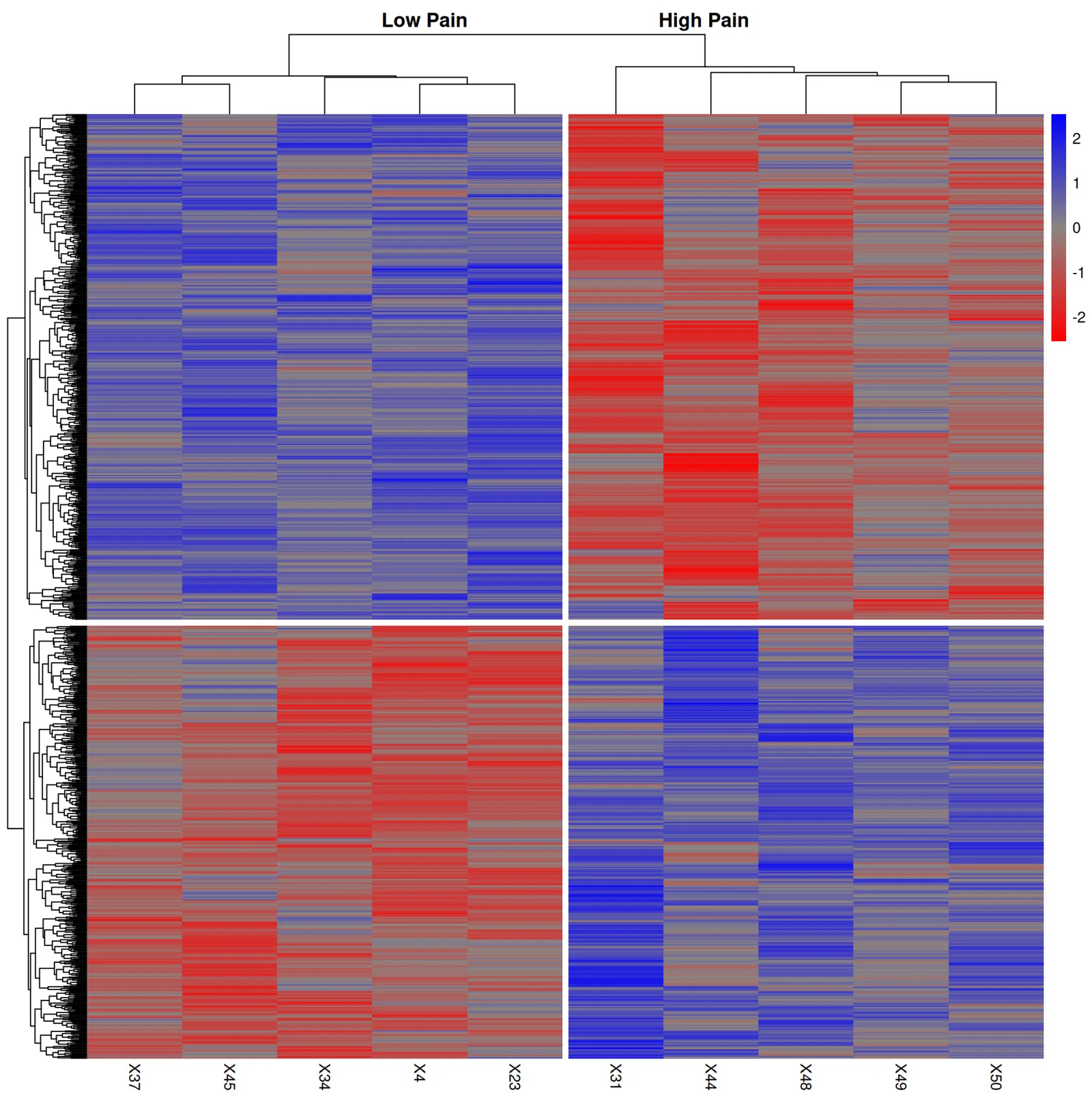
Differentially Expressed Proteins Heatmap. Heatmap of differentially expressed proteins (n-1800; FDR < 0.05). Unsupervised hierarchical clustering of samples and proteins. Columns represent participants; rows represent proteins. (blue = low expression proteins in high pain patients; red = High expression proteins in low pain patients), segregated by pain status.

**Figure 4:**
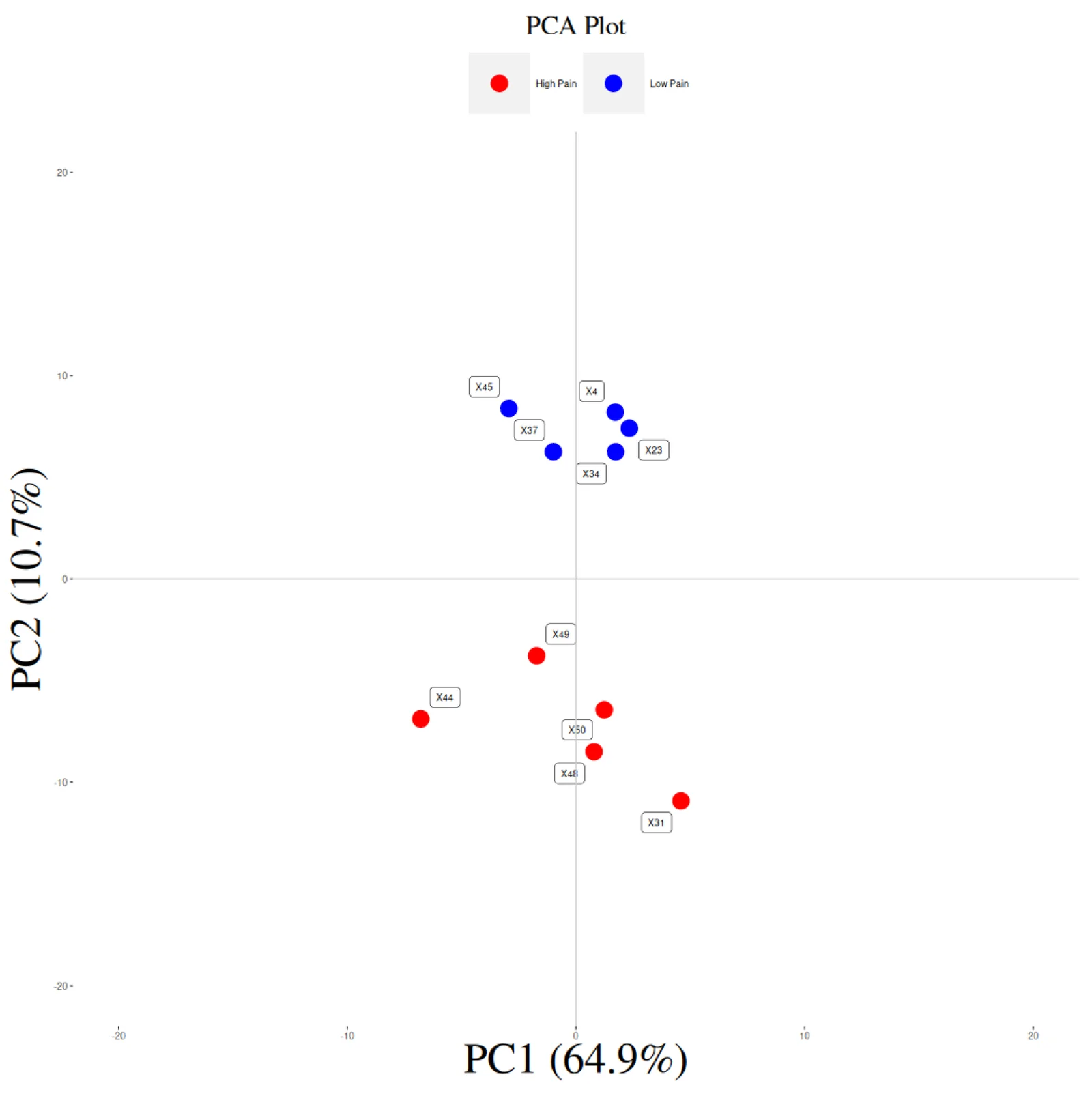
Differentially Expressed Proteins PCA. A principal component analysis (PCA) plot illustrating the segregation of samples based on protein expression profiles. Principal component 1 (PC1) explains 64.9% of the variance and principal component 2 (PC2) explains 10.7% of the variance. Samples cluster according to pain status, with high pain samples shown in red and low pain samples shown in blue. Each point represents an individual sample, demonstrating clear group separation driven by global proteomic differences.

**Figure 5:**
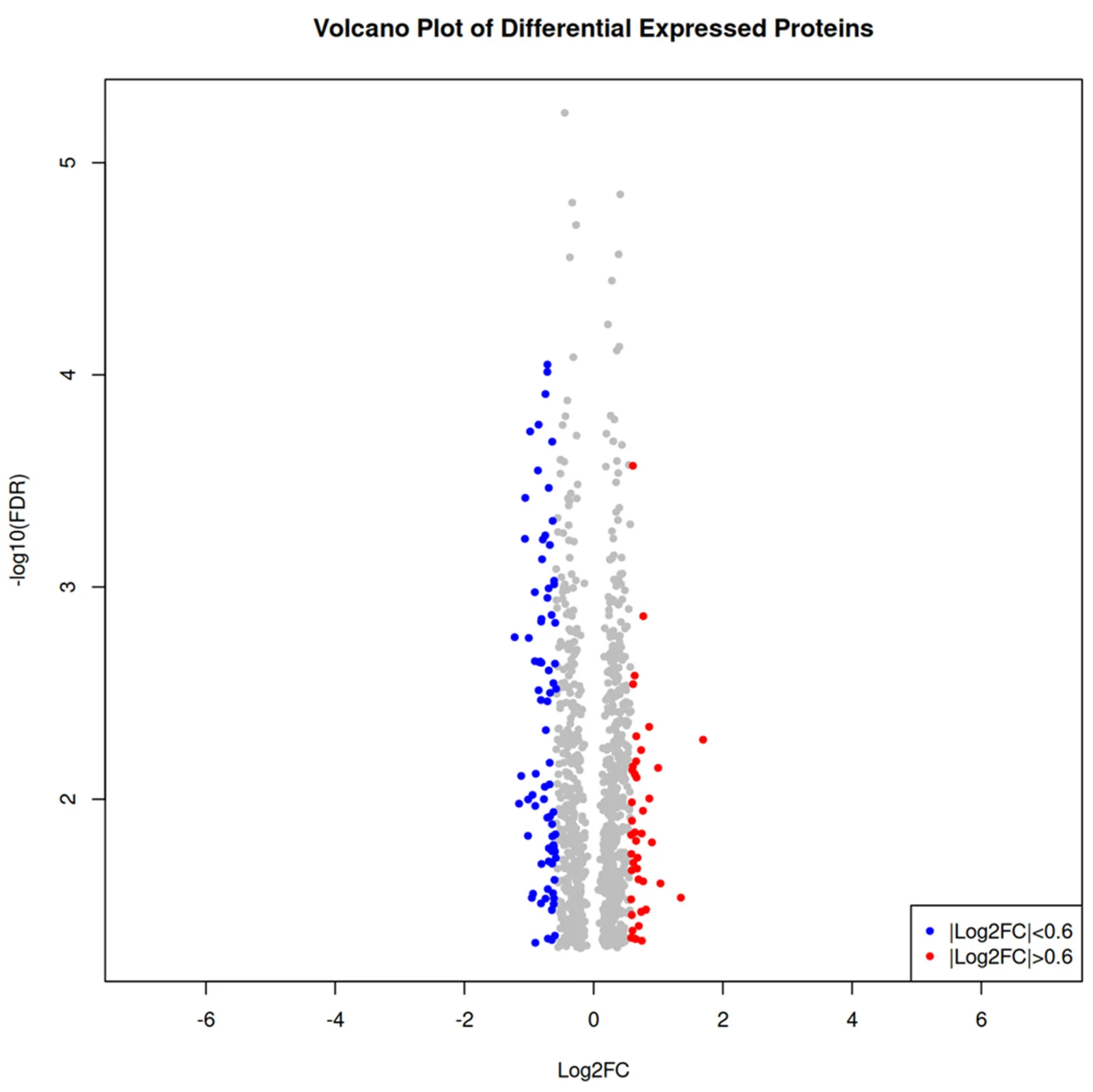
Volcano Plot of Differentially Expressed Proteins. FDR < 0.05. Proteins that increase with pain are red. Proteins that decresase with pain are blue.

A total of 4196 proteins were identified from platelet samples in adults with SCD. Differential analysis between the high and low pain phenotypes revealed a large proteomic signature (1027 proteins, FDR < 0.05) (Figure 3 and Figure 4, Supplemental Tables S1 and S2). Volcano plot analysis identified numerous proteins meeting significance criteria (FDR < 0.05 and |log2FC| > 0.56), with biologically meaningful changes in abundance (Figure 5). Proteins increased in the high-pain group were represented in red, whereas proteins decreased with pain were represented in blue, demonstrating a broad distribution of differentially expressed proteins associated with the pain phenotype. In the pathway analyses, contrary to expectations, canonical pain pathways were not enriched. Instead, pathways related to ATP metabolism, mitochondrial function, mitophagy, and neurodegeneration were prominently represented (Supplementary Tables S1–S11).

**Figure 6:**
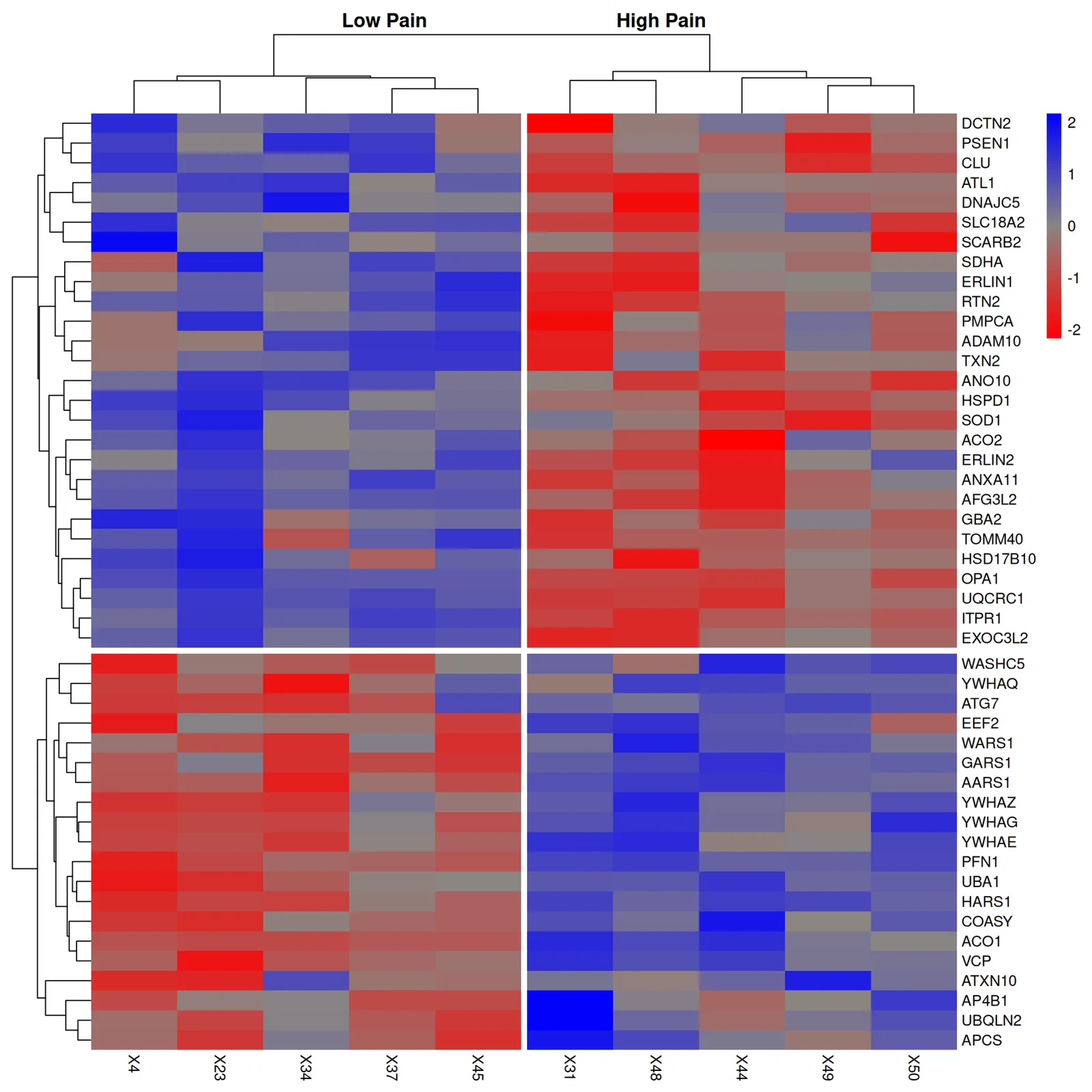
Neurodegeneration Heatmap. The heatmap shows unsupervised hierarchical clustering of significantly altered proteins across low pain (**left**) and high pain (**right**) samples. Rows represent proteins and columns represent individual samples. Protein abundance is displayed as row scaled expression values, with blue indicating lower and red indicating higher relative expression. Distinct clustering patterns highlight differential regulation of mitochondrial, vesicular trafficking, and protein homeostasis pathways between pain groups.

These findings demonstrate that unsupervised clustering methods effectively separated participants according to pain profiles and corresponding proteomic signatures, supporting the presence of biologically and clinically distinct pain phenotypes within the SCD cohort (Figure 1, Figure 2, Figure 3, Figure 4, Figure 5 and Figure 6).

### 3.3. Platelet Proteome Distinguishes Pain Groups

Principal component analysis (PCA) and differentially expressed proteins heatmap demonstrated clear separation between high- and low-pain phenotypes based on platelet proteomic profiles (Figure 4). Principal component 1 (PC1) accounted for 64.9% of the variance and distinctly separated high-pain participants from low-pain participants, while principal component 2 (PC2) explained an additional 10.7% of the variance, reflecting within-group heterogeneity. High-pain samples clustered predominantly within the lower quadrants of the PCA space, whereas low-pain samples clustered separately in the upper quadrants, indicating strong differences in global protein expression patterns between pain phenotypes.

### 3.4. Neurodegeneration

Proteomic analyses identified significant alterations in proteins associated with neurodegenerative pathways (Supplemental Table S1, Figure 6). Proteins significantly increased at a fold change ≥ +1.5 included PFN1 (Profilin 1), UBQLN2 (Ubiquilin 2), APCS (Amyloid P Component, Serum), and CLU (Clusterin). Additional proteins increased at a fold change ≥ +1.2 included ATG7 (Autophagy Related 7), EEF2 (Eukaryotic Translation Elongation Factor 2), WARS1 (Tryptophanyl-tRNA Synthetase 1), GARS1 (Glycyl-tRNA Synthetase 1), AARS1 (Alanyl-tRNA Synthetase 1), YWHAZ (Tyrosine 3-Monooxygenase/Tryptophan 5-Monooxygenase Activation Protein Zeta), YWHAG (Tyrosine 3-Monooxygenase/Tryptophan 5-Monooxygenase Activation Protein Gamma), YWHAE (Tyrosine 3-Monooxygenase/Tryptophan 5-Monooxygenase Activation Protein Epsilon), UBA1 (Ubiquitin Like Modifier Activating Enzyme 1), HARS1 (Histidyl-tRNA Synthetase 1), ACO1 (Aconitase 1), VCP (Valosin Containing Protein), and AP4B1 (Adaptor Related Protein Complex 4 Subunit Beta 1).

**Figure 7:**
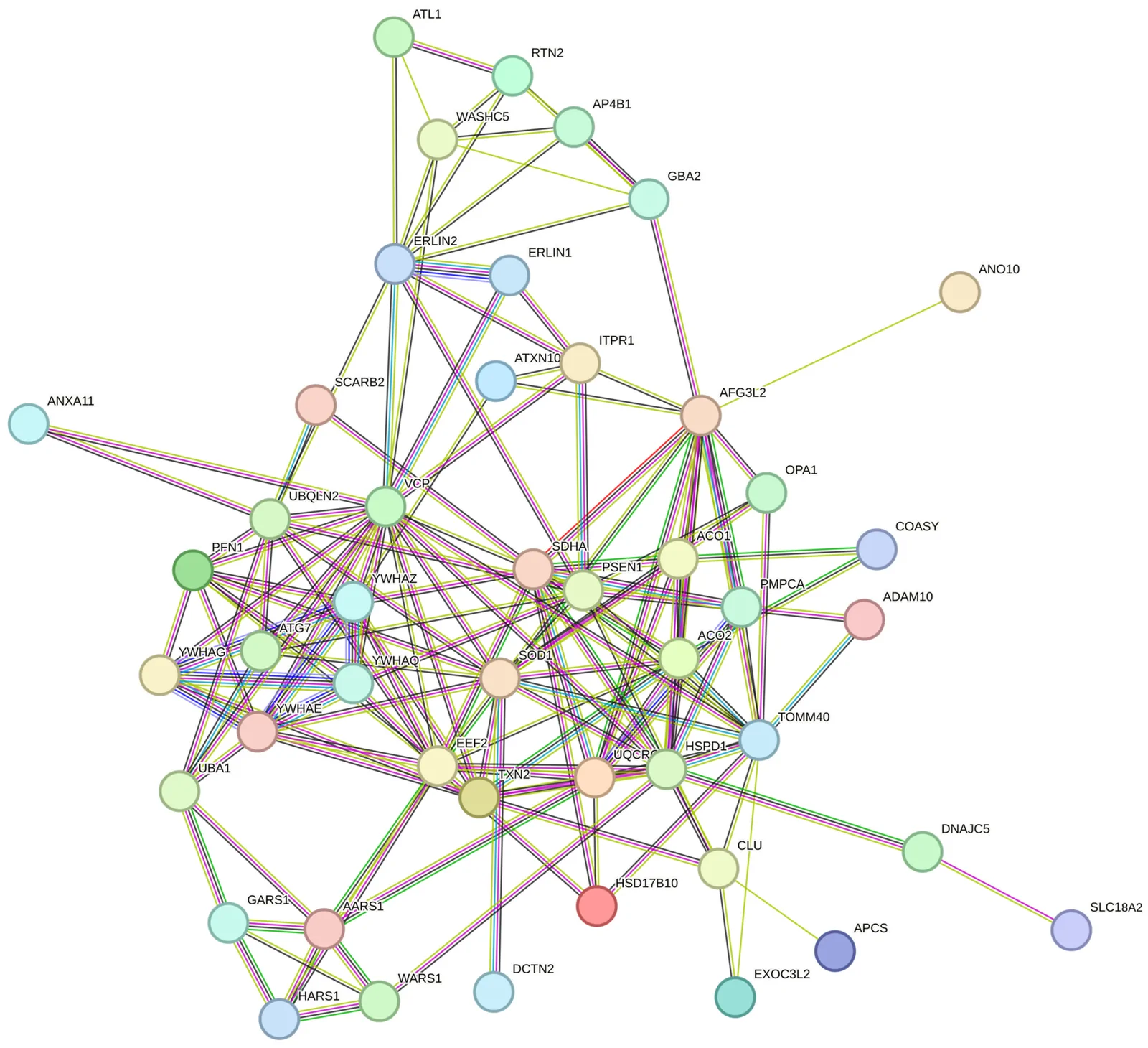
Neurodegeneration PPI. Network visualization of proteins associated with neurodegeneration related pathways. Highly interconnected hubs involved in mitochondrial function, protein homeostasis, trafficking, cytoskeletal regulation, and stress responses highlight coordinated networks linked to pain associated neurodegenerative and neuroadaptive processes.

Proteins significantly reduced at a fold change ≥ −1.2 included PSEN1 (Presenilin 1), DNAJC5 (DnaJ Heat Shock Protein Family Member C5), SLC18A2 (Solute Carrier Family 18 Member A2), SCARB2 (Scavenger Receptor Class B Member 2), RTN2 (Reticulon 2), ADAM10 (ADAM Metallopeptidase Domain 10), TXN2 (Thioredoxin 2), ANO10 (Anoctamin 10), HSPD1 (Heat Shock Protein Family D Member 1), SOD1 (Superoxide Dismutase 1), ANXA11 (Annexin A11), AFG3L2 (AFG3 Like Matrix AAA Peptidase Subunit 2), HSD17B10 (Hydroxysteroid 17-Beta Dehydrogenase 10), UQCRC1 (Ubiquinol-Cytochrome C Reductase Core Protein 1), and EXOC3L2 (Exocyst Complex Component 3 Like 2) (Supplemental Table S2, Figure 6 and Figure 7).

### 3.5. Central Nervous System

Analysis of central nervous system-related proteins demonstrated substantial alterations associated with the pain phenotype in SCD (Supplemental Table S2). Proteins significantly increased at a fold change ≥ +1.5 included CASP3 (Caspase 3), IDH1 (Isocitrate Dehydrogenase [NADP] Cytoplasmic), COL4A2 (Collagen Alpha 2[IV] Chain), UBQLN2 (Ubiquilin 2), and APCS (Serum Amyloid P Component). Proteins significantly reduced at a fold change ≥ −1.5 included HSPE1 (10 kDa Heat Shock Protein, Mitochondrial [Hsp10]), DST (Dystonin), SLC66A2 (Solute Carrier Family 66 Member 2), MT-ND6 (NADH Ubiquinone Oxidoreductase Chain 6), NDUFB9 (NADH Dehydrogenase [Ubiquinone] 1 Beta Subcomplex Subunit 9), and CLU (Clusterin).

**Figure 8:**
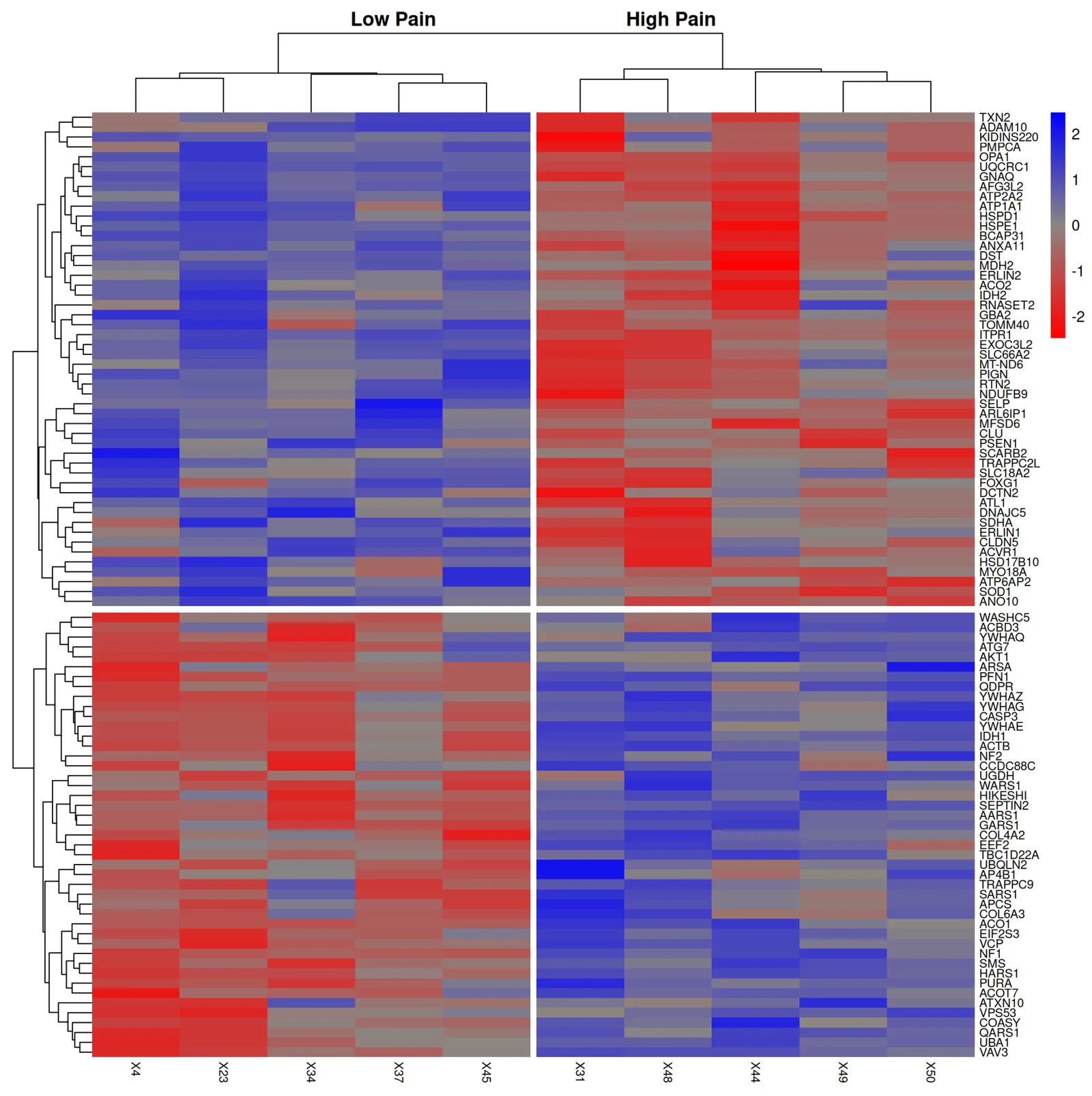
Central nervous system heatmap. Unsupervised hierarchical clustering of central nervous system proteins that were significantly differentially expressed between low pain (**left**) and high pain (**right**) groups. Rows represent proteins and columns represent individual samples. Protein abundance is shown as row-scaled expression values, with red indicating higher relative expression and blue indicating lower relative expression. The heatmap reveals distinct clustering of samples by pain status and highlights coordinated changes in proteins involved in mitochondrial function, synaptic/vesicular trafficking, cytoskeletal organization, and protein homeostasis.

**Figure 9:**
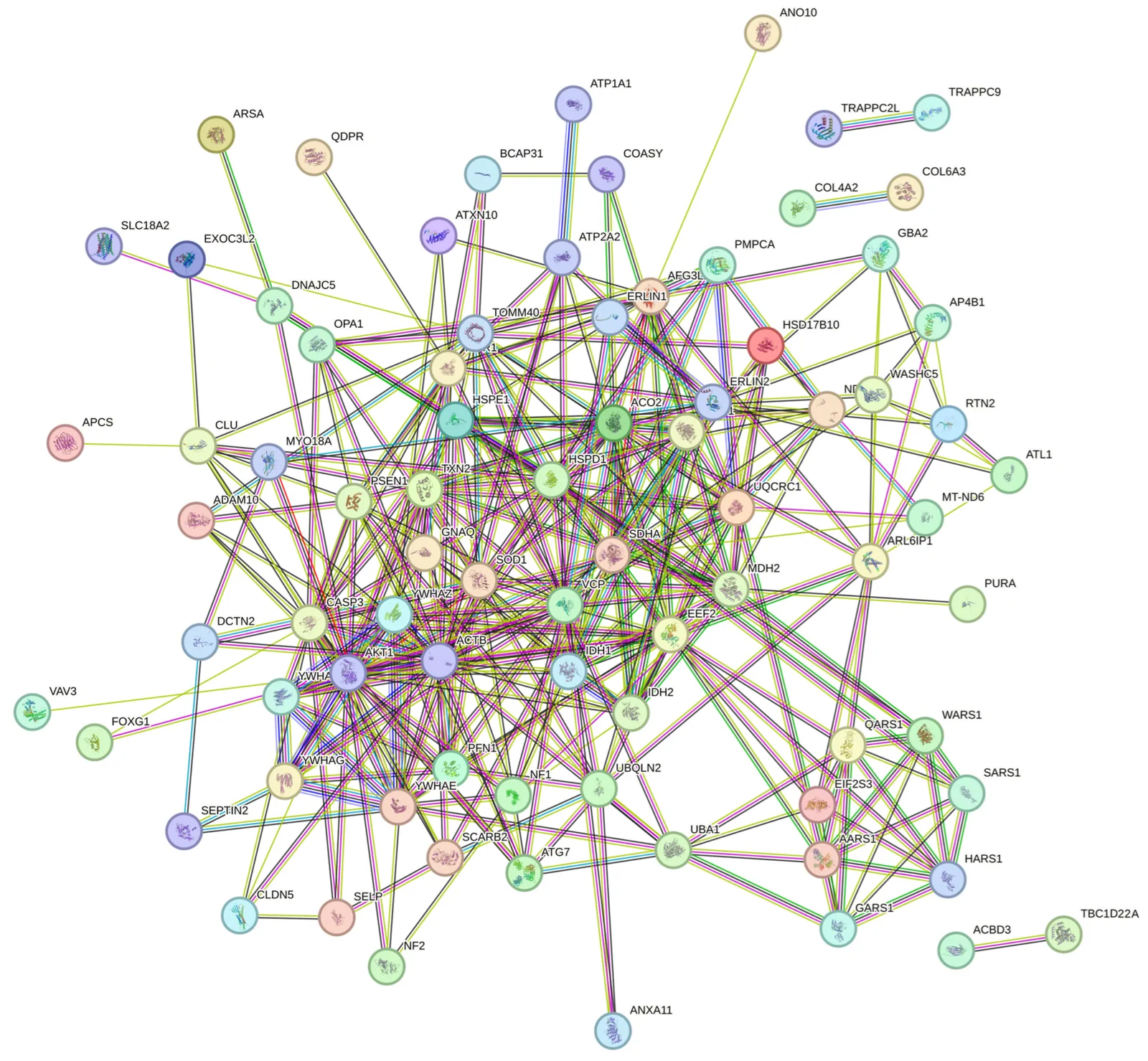
Central nervous system PPI. Network visualization of significantly altered central nervous system related proteins. Highly connected hubs linked to mitochondrial function, protein homeostasis, trafficking, cytoskeletal organization, and stress responses highlight coordinated networks underlying pain-associated CNS dysfunction and neuroadaptation.

Additional proteins increased at a fold change ≥ +1.2 included proteins involved in cellular signaling, autophagy, translational regulation, and proteostasis, including YWHAQ (14-3-3 Protein Theta), ATG7 (Autophagy Related Protein 7), AKT1 (RAC Alpha Serine/Threonine Protein Kinase), PFN1 (Profilin 1), WARS1 (Tryptophan tRNA Ligase, Cytoplasmic), AARS1 (Alanine tRNA Ligase, Cytoplasmic), GARS1 (Glycine tRNA Ligase), EEF2 (Elongation Factor 2), EIF2S3 (Eukaryotic Translation Initiation Factor 2 Subunit 3), VCP (Transitional Endoplasmic Reticulum ATPase/Valosin Containing Protein), NF1 (Neurofibromin), HARS1 (Histidine tRNA Ligase, Cytoplasmic), and UBA1 (Ubiquitin Like Modifier Activating Enzyme 1) (Supplemental Table S2, Figure 8 and Figure 9).

Proteins reduced at a fold change ≥ −1.2 included several proteins involved in mitochondrial regulation, vesicular trafficking, neuronal signaling, and membrane integrity, including TXN2 (Thioredoxin, Mitochondrial), ADAM10 (Disintegrin and Metalloproteinase Domain Containing Protein 10), AFG3L2 (AFG3 Like Protein 2), HSPD1 (60 kDa Heat Shock Protein, Mitochondrial [Hsp60]), ANXA11 (Annexin A11), MDH2 (Malate Dehydrogenase, Mitochondrial), RTN2 (Reticulon 2), PSEN1 (Presenilin 1), SCARB2 (Lysosomal Integral Membrane Protein 2 [LIMP-2]), SLC18A2 (Vesicular Monoamine Transporter 2 [VMAT2]), DNAJC5 (DnaJ Homolog Subfamily C Member 5 [CSPα]), CLDN5 (Claudin 5), and ANO10 (Anoctamin 10) (Supplemental Table S2, Figure 8 and Figure 9).

IL-12, IL-17, and IL-21 were detected, but their abundance did not differ significantly between groups; IL-18 was the only interleukin that demonstrated a significant change. Nevertheless, pathway analysis identified 47 significantly altered proteins associated with the positive regulation of cytokine production, supporting broader alteration of cytokine-related processes rather than a conventional cytokine-dominant inflammatory response (Supplemental Table S1).

### 3.6. Mitophagy

**Figure 10:**
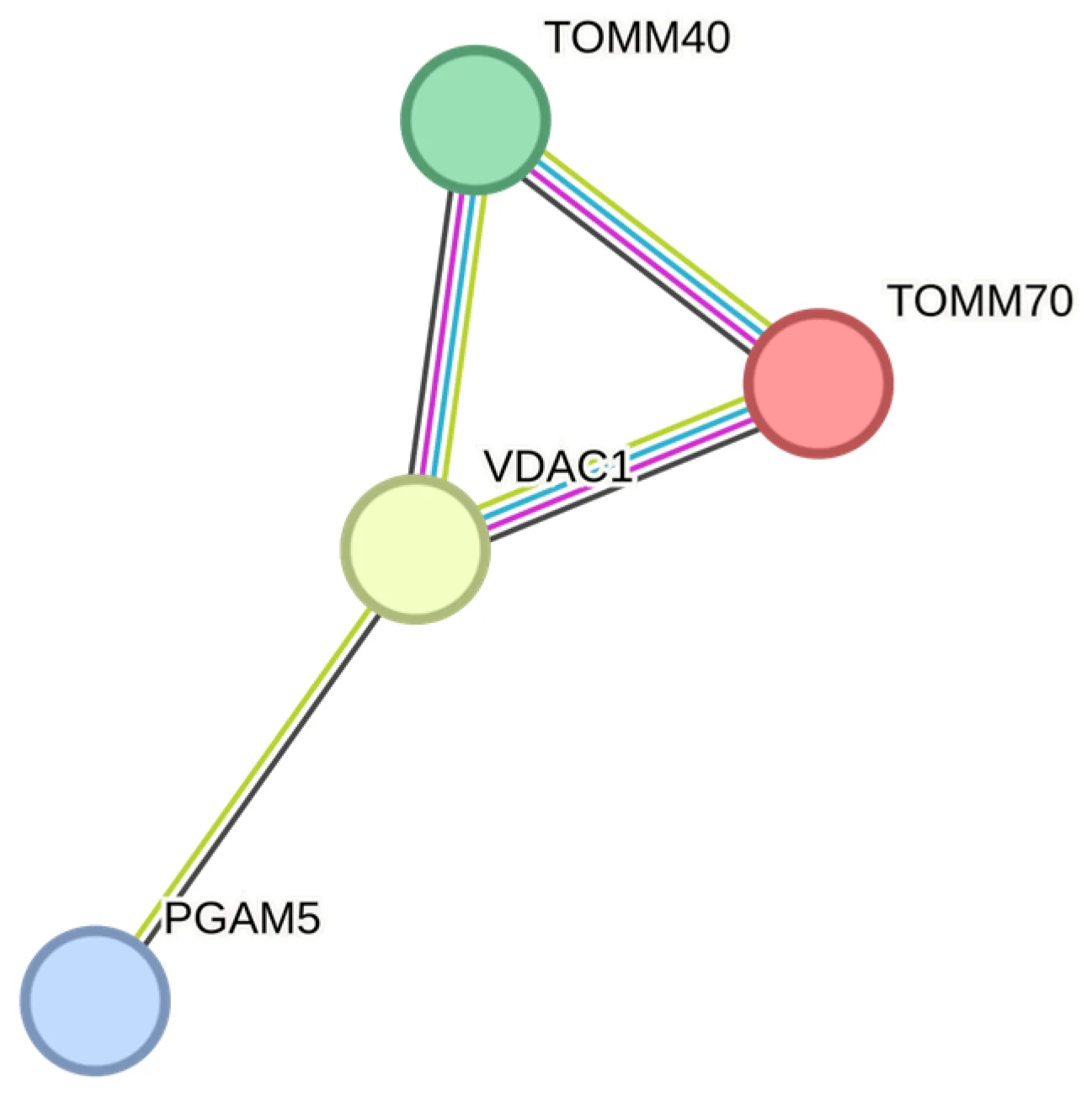
Mitophagy related protein–protein interaction (PPI) network. Nodes represent proteins and edges indicate interactions. VDAC1 serves as a central hub connecting TOMM40, TOMM70, and PGAM5, highlighting coordinated roles in mitochondrial import and mitophagy.

At the fold change ≥ −1.2 threshold, reduced expression was observed for TOMM70 (Translocase of Outer Mitochondrial Membrane 70) and VDAC1 (Voltage Dependent Anion Channel 1), both involved in mitochondrial membrane integrity and mitochondrial quality control (Supplemental Table S2, Figure 10).

### 3.7. Respiratory Electron Transport ATP Synthesis by Chemiosmotic Coupling and Heat Production by Uncoupling Proteins

Proteins involved in mitochondrial respiratory electron transport and ATP synthesis demonstrated broad downregulation (Supplemental Table S1). Proteins significantly reduced at a fold change ≥ −1.5 included NDUFB9 (NADH:Ubiquinone Oxidoreductase Subunit B9), NDUFV2 (NADH:Ubiquinone Oxidoreductase Core Subunit V2), COX6C (Cytochrome C Oxidase Subunit 6C), NDUFB11 (NADH:Ubiquinone Oxidoreductase Subunit B11), NDUFB8 (NADH:Ubiquinone Oxidoreductase Subunit B8), MT-ND6 (Mitochondrially Encoded NADH Dehydrogenase 6), and NDUFB1 (NADH:Ubiquinone Oxidoreductase Subunit B1) (Supplemental Tables S1 and S2).

**Figure 11:**
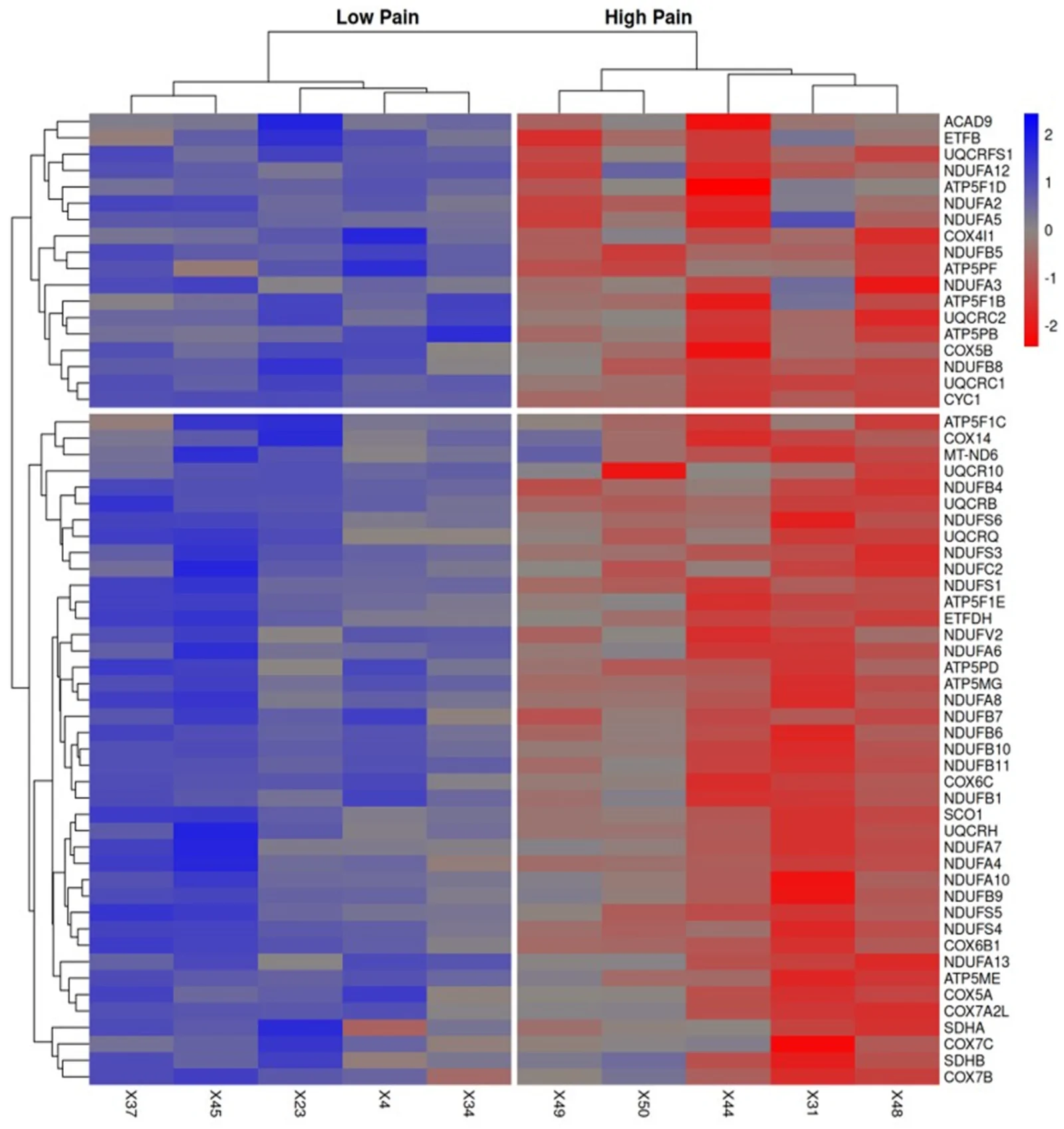
Respiratory Electron Transport Heatmap. Unsupervised hierarchical clustering of proteins involved in mitochondrial electron transport, oxidative phosphorylation, ATP synthesis, and chemiosmotic coupling across low pain (**left**) and high pain (**right**) samples. Rows represent individual mitochondrial proteins and columns represent samples. Protein abundance is shown as row-scaled expression values, with red indicating higher relative expression and blue indicating lower relative expression. The heatmap demonstrates coordinated downregulation of multiple respiratory chain complex subunits in low pain samples and relative upregulation in high pain samples, highlighting pain-associated alterations in mitochondrial energy metabolism.

**Figure 12:**
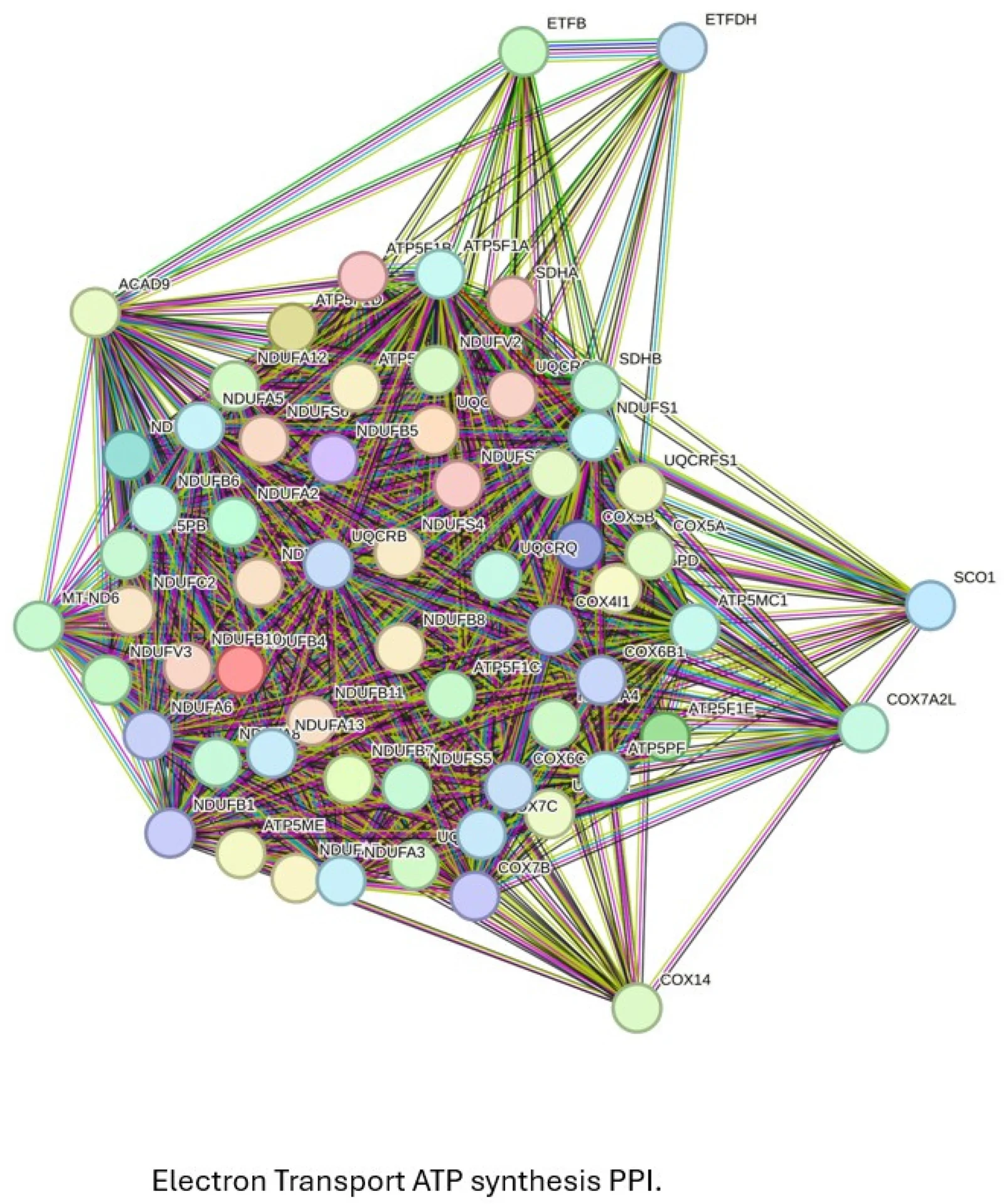
Respiratory Electron Transport ATP PPI. Protein–protein interaction network of mitochondrial electron transport chain complexes (I–IV) and ATP synthase (V), highlighting dense connectivity among NDUF, UQCR, COX, and ATP5 subunits. Peripheral proteins (e.g., ETFB, ETFDH, SCO1) indicate auxiliary roles in electron transfer and complex assembly, illustrating coupling of ATP production and potential uncoupling-related thermogenesis.

Additional proteins reduced at a fold change ≥ −1.2 included COX7C (Cytochrome C Oxidase Subunit 7C), NDUFA13 (NADH:Ubiquinone Oxidoreductase Subunit A13), NDUFB4 (NADH:Ubiquinone Oxidoreductase Subunit B4), SCO1 (Synthesis of Cytochrome C Oxidase 1), ATP5PF (ATP Synthase Peripheral Stalk Subunit F6), UQCRFS1 (Ubiquinol-Cytochrome C Reductase Rieske Iron-Sulfur Polypeptide 1), SDHB (Succinate Dehydrogenase Complex Iron Sulfur Subunit B), UQCRC1 (Ubiquinol-Cytochrome C Reductase Core Protein 1), NDUFB5 (NADH:Ubiquinone Oxidoreductase Subunit B5), NDUFA6 (NADH:Ubiquinone Oxidoreductase Subunit A6), UQCRQ (Ubiquinol-Cytochrome C Reductase Complex III Subunit VII), COX7B (Cytochrome C Oxidase Subunit 7B), NDUFA2 (NADH:Ubiquinone Oxidoreductase Subunit A2), NDUFC2 (NADH:Ubiquinone Oxidoreductase Subunit C2), ATP5MG (ATP Synthase Membrane Subunit G), COX6B1 (Cytochrome C Oxidase Subunit 6B1), ATP5F1D (ATP Synthase F1 Subunit Delta), NDUFA3 (NADH:Ubiquinone Oxidoreductase Subunit A3), NDUFA7 (NADH:Ubiquinone Oxidoreductase Subunit A7), NDUFS4 (NADH:Ubiquinone Oxidoreductase Core Subunit S4), CYC1 (Cytochrome C1), COX5A (Cytochrome C Oxidase Subunit 5A), ATP5PD (ATP Synthase Peripheral Stalk Subunit D), NDUFB7 (NADH:Ubiquinone Oxidoreductase Subunit B7), UQCRH (Ubiquinol-Cytochrome C Reductase Hinge Protein), NDUFB6 (NADH:Ubiquinone Oxidoreductase Subunit B6), ATP5ME (ATP Synthase Membrane Subunit E), COX7A2L (Cytochrome C Oxidase Subunit 7A2 Like), UQCR10 (Ubiquinol-Cytochrome C Reductase Complex III Subunit X), NDUFA8 (NADH:Ubiquinone Oxidoreductase Subunit A8), NDUFB10 (NADH:Ubiquinone Oxidoreductase Subunit B10), UQCRB (Ubiquinol-Cytochrome C Reductase Binding Protein), COX14 (Cytochrome C Oxidase Assembly Factor COX14), NDUFS6 (NADH:Ubiquinone Oxidoreductase Subunit S6), ATP5F1E (ATP Synthase F1 Subunit Epsilon), and NDUFA10 (NADH:Ubiquinone Oxidoreductase Subunit A10) (Supplemental Table S2, Figure 11 and Figure 12).

### 3.8. Glutathione Metabolic Process

**Figure 13:**
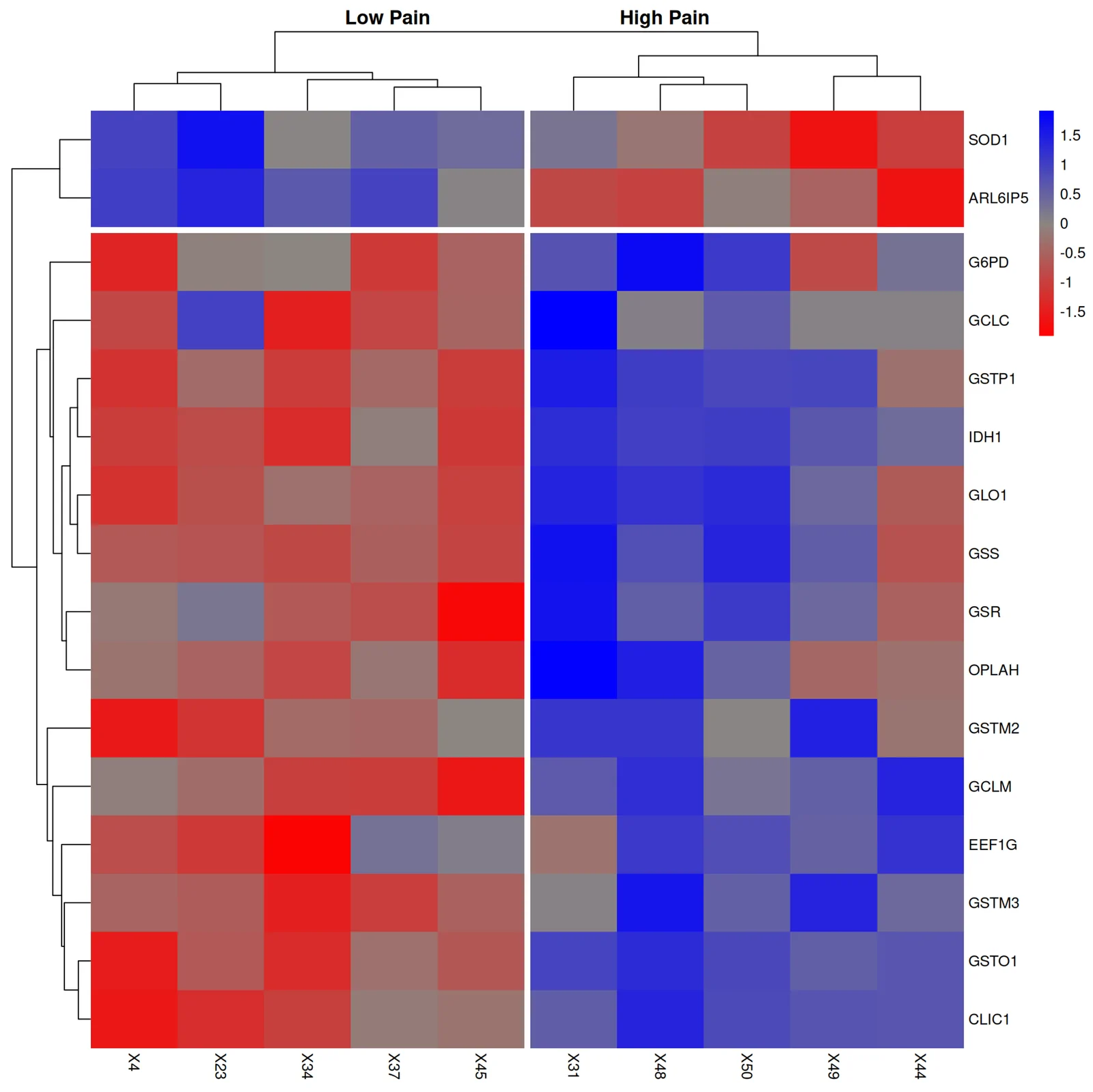
Glutathione metabolic process heatmap. Unsupervised hierarchical clustering of proteins involved in glutathione metabolism and redox regulation in low pain (**left**) and high pain (**right**) samples. Row-scaled expression values show increased abundance of glutathione synthesis and detoxification enzymes in high pain samples, indicating enhanced oxidative stress responses.

**Figure 14:**
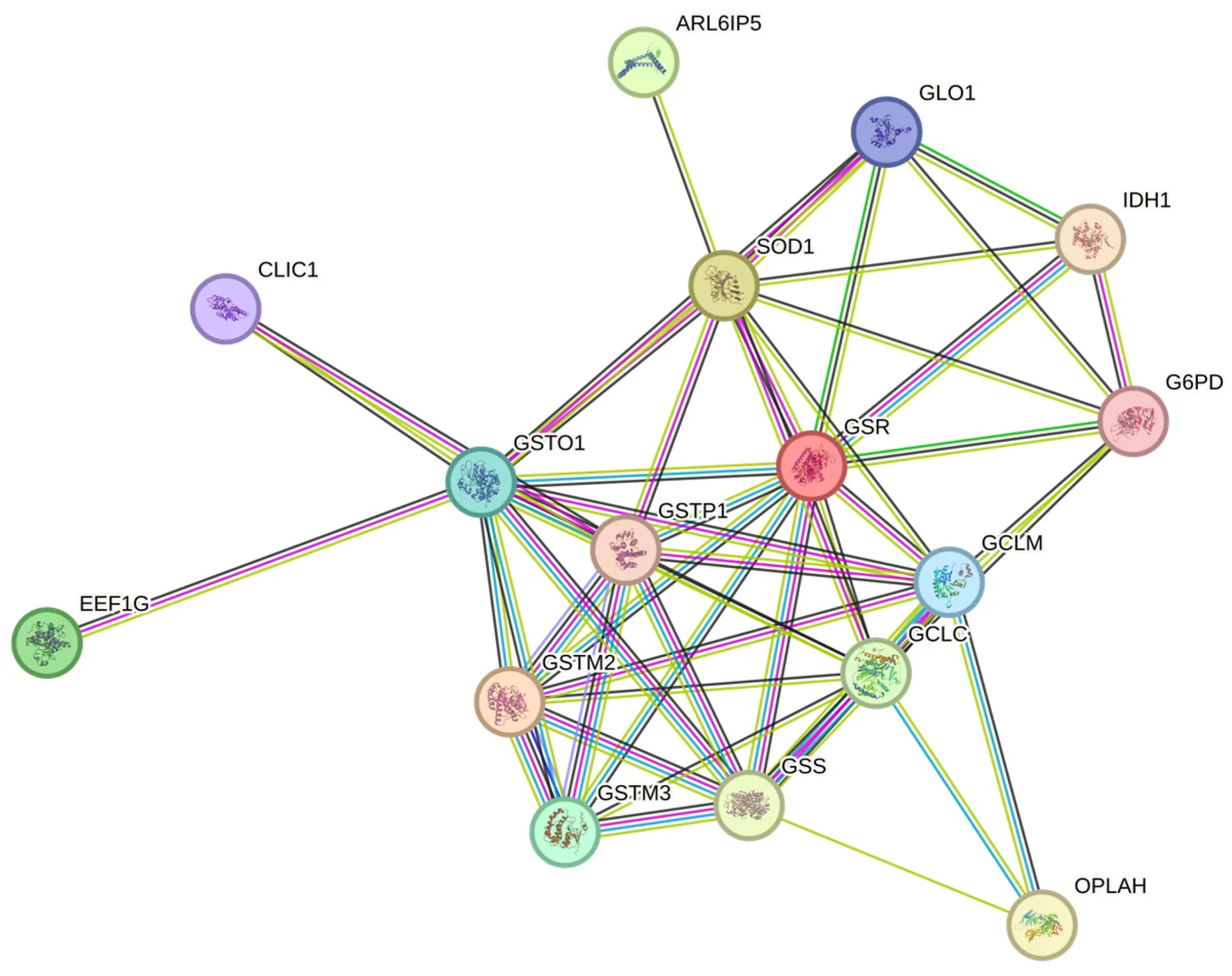
Glutathione metabolic process PPI. Compact PPI network highlighting differentially expressed proteins involved in glutathione synthesis, recycling, and redox regulation. Nodes represent enzymes in antioxidant and detoxification pathways, with dense connectivity indicating coordinated regulation of oxidative stress responses.

Proteins involved in glutathione metabolism were significantly increased. Proteins meeting the fold change ≥ +1.5 threshold included GSR (Glutathione Reductase), GSTP1 (Glutathione S-Transferase Pi 1), and GSS (Glutathione Synthetase). No glutathione-related proteins were significantly reduced (Supplemental Table S2, Figure 13 and Figure 14).

### 3.9. Glycolysis Pathway

**Figure 15:**
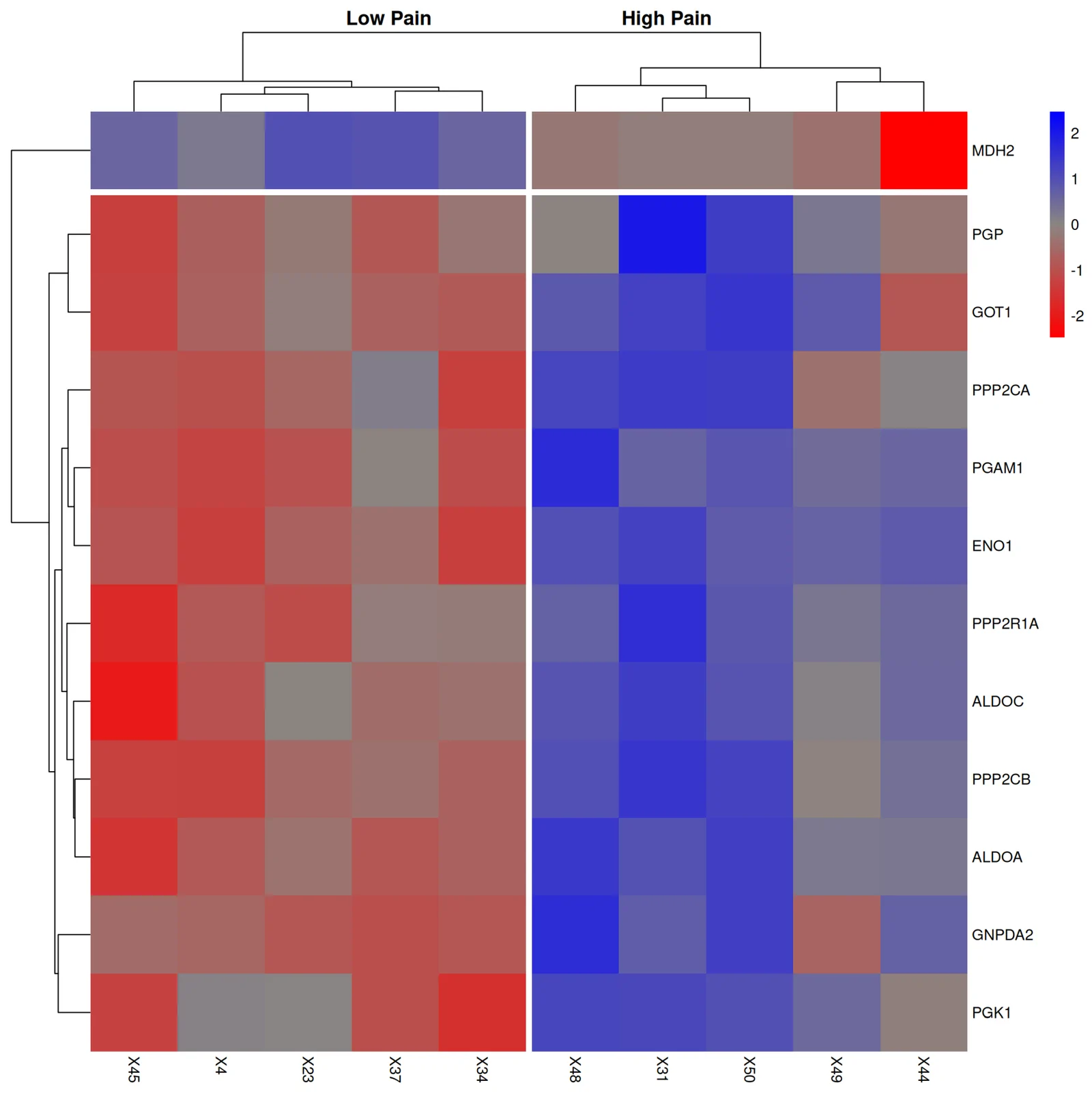
Glucose metabolism heatmap. Unsupervised hierarchical clustering of differentially expressed glucose metabolism–related proteins in low pain (**left**) and high pain (**right**) samples. Row-scaled expression values show coordinated upregulation of key glycolytic enzymes and regulators in the high pain group, indicating pain-associated metabolic remodeling.

**Figure 16:**
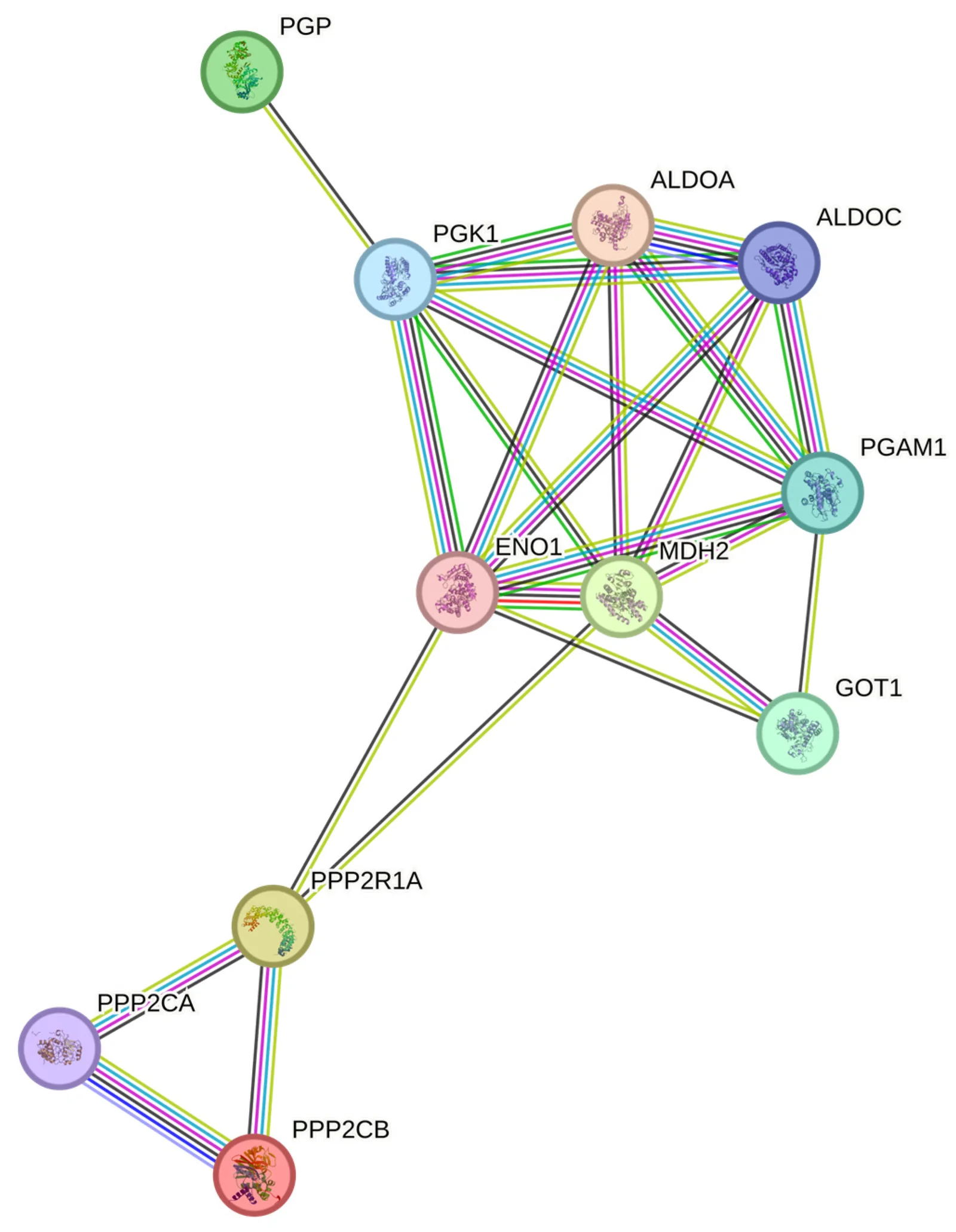
Glucose metabolism PPI associated proteins. Protein—interaction network of significantly altered glucose metabolism–related proteins. Nodes represent metabolic enzymes and regulatory proteins, and edges denote known or predicted interactions, highlighting coordinated regulation of glycolysis and associated metabolic pathways in relation to pain.

Glycolytic pathway proteins demonstrated increased expression. Proteins significantly elevated at a fold change ≥ +1.5 included PGK1 (Phosphoglycerate Kinase 1) and ENO1 (Enolase 1). ENO1 demonstrated a fold change of 1.642 (FDR = 9.68699 × 10^−5^). No glycolytic proteins were significantly reduced (Supplemental Table S2, Figure 15 and Figure 16).

## 4. Discussion

In this study, we used platelet proteomic profiling to investigate molecular signatures associated with pain phenotypes in adults with SCD. Rather than enrichment of canonical nociceptive or inflammatory pain pathways, our results revealed robust and coordinated alterations in mitochondrial function, oxidative stress regulation, proteostasis, and neurodegenerative signaling that strongly distinguished high- and low-pain phenotypes. Unsupervised clustering and multivariate analyses demonstrated that platelet proteomic profiles segregated individuals by pain burden in a manner concordant with clinical measures of neuropathic pain, suggesting that systemic cellular stress responses, rather than traditional pain signaling pathways, may underlie chronic pain heterogeneity in SCD. Collectively, these findings support a model in which mitochondrial dysfunction, oxidative stress, degenerative changes, impaired protein quality control, and metabolic reprogramming contribute to pain severity in SCD and position platelets as a biologically informative and accessible window into these processes.

Although platelets lack a nucleus, their proteome is dynamic. Platelet proteins may be inherited from megakaryocytes, translated from retained megakaryocyte-derived mRNAs, synthesized within mitochondria, or taken up from the circulation. Thus, interpatient differences in protein abundance may reflect variation in megakaryocyte programming, disease-related signaling, platelet activation, protein uptake and turnover, and post-translational modifications [54].

Protein expression levels and modifications, along with the impact of psychosocial and neurocognitive factors, remain under-investigated in SCD. Proteomic research is beginning to reveal how mitochondrial dysfunction and neurodegeneration pathways may shape pain perception in individuals with SCD.

In this study, we used platelet proteomics to explore molecular correlates of pain in individuals with SCD. While our initial hypothesis focused on pain-associated pathways, our analysis revealed a striking and unexpected enrichment of pathways related to neurodegeneration, cognitive dysfunction, and mitochondrial dysregulation. These findings suggest that platelets may reflect systemic and neurological alterations in SCD that extend beyond their traditional roles in hemostasis and inflammation [27,55,56], and relate to the overall pain experience.

The proteins listed in Supplemental Table S1 include 81 proteins with FDR < 0.05 increasing over 1.5-fold and 38 proteins with FDR < 0.05 down-regulated over 1.5-fold in self-reported “high-pain” versus “low-pain” phenotypes in SCD. Supplemental Table S1 includes 318 proteins that increase at least 1.2-fold and 348 proteins that decrease at least 1.2-fold with FDR < 0.05. Analysis of both proteomic tables suggest that platelets in high pain SCD patients carry distinct signatures for defective mitochondrial electron transport, oxidative phosphorylation, and mitophagy, and increased and decreased proteins that mirror systemic injury: neurodegeneration, neuropathic pain, and vaso-occlusive crises (VOCs). The data indicates that platelets from high-pain patients are enriched with proteins typically associated with central nervous system (CNS) injury and neurodegenerative pathways. Upregulated proteins such as UBQLN2 (Ubiquilin-2) and APCS (Amyloid P Component, Serum) are established markers of neurodegeneration [57,58] Their elevation in the platelet proteome provides evidence of neurological injury likely caused by chronic silent cerebral infarcts (SCIs) and repetitive hypoxic stress from vaso-oclusion.

The downregulation of mitochondrial respiratory chain Complex I and Complex V subunits (e.g., NDUFB9 (NADH:Ubiquinone Oxidoreductase Subunit B9), NDUFV2 (NADH:Ubiquinone Oxidoreductase Core Subunit V2), ATP5F1 (ATP Synthase Peripheral Stalk Subunit B)) points to a systemic state of mitochondrial dysfunction and oxidative stress [59] This impairment is a hallmark of neurodegenerative processes and suggests that the energy-producing capacity of cells is severely compromised in high-pain individuals. The presence of specific metabolic and signaling proteins indicates a transition from acute nociceptive pain to chronic neuropathic pain [60] The increased expression of proteins involved in tRNA aminoacylation (e.g., WARS1 (Tryptophanyl-tRNA synthetase 1), GARS1 (Glycyl tRNA synthetase)) and inflammatory mediators (HSP90AB4P (Heat Shock Protein 90-beta 4P), and CASP3 (Caspase-3) suggest that platelets are responding to, and perhaps contributing to, the neuroinflammatory environment that drives neuropathic pain. These protein signatures align with the clinical phenotype of central sensitization, where the nervous system remains in a persistent state of high reactivity, making patients more susceptible to chronic pain even in the absence of an active VOC [61,62] While vaso-occlusive crises (VOCs) are traditionally viewed as an acute event, the platelet proteome reveals an increase expression of proteins (e.g., SERPINA7 and SERPINB6 (Thyroxine-Binding Globulin, Placental Thrombin Inhibitor) that indicate a chronic pro-thrombotic and inflammatory state in high-pain patients. Upregulated proteins such as CA2 (Carbonic Anhydrase 2) and members of the actin cytoskeleton pathway (e.g., PFN1 (Profilin 1) ACTN2 (Alpha-actinin-2), and CFL1 (Cofilin-1), suggest that platelets in high-pain patients are hyper-reactive and primed for adhesion, facilitating the vaso-occlusion that characterize VOCs [63] The upregulation of glycolytic enzymes (e.g., PGK1; Phosphoglycerate Kinase 1), ENO1 (Enolase 1) (see Supplementary Tables S1 and S7, Reactome) suggests a metabolic shift to anaerobic pathways, a common cellular response to the chronic ischemia-reperfusion injury seen in SCD [64] Although platelets contain only a small number of mitochondria, they retain functional mitochondrial quality control programs, including mitophagy [65] Platelet mitophagy has been shown to occur in platelets and, in vivo, helps maintain mitochondrial quality while shaping platelet activation and lifespan [66] Red blood cells and platelets often fail to properly clear damaged mitochondria, a process known as mitophagy. As demonstrated in Supplementary Table S1, diminished mitophagy proteins (fold change of at least 1.2-fold) in the high pain cohort (TOMM70 (Translocase of Outer Mitochondrial Membrane 70), VDAC1 (Voltage-Dependent Anion Channel 1) lead to the retention of dysfunctional mitochondria. Without sufficient TOMM70 and VDAC1, the kinase PINK1 (PTEN-induced kinase 1) cannot stabilize on the mitochondrial outer membrane to recruit the protein Parkin, initiating mitophagy. Thus, a reduction in TOMM70 limits the efficient recruitment of the E3 ubiquitin ligase Parkin, preventing the ubiquitin tagging of the organelle for destruction. As VDAC1 is a primary target for Parkin mediated polyubiquitination, diminished VDAC1 levels cause reduction of ubiquitin tagged damaged mitochondria removal. VDAC1 is also involved in the recruitment of the phagophore membrane to the surface of the damaged mitochondria. All are vital for cellular homeostasis and organelle degradation [67,68] These retained mitochondria release cell-free mitochondrial DNA (mtDNA) that activates neutrophils and promotes the systemic inflammatory state underlying neuropathic pain [69] The decrease in mitochondrial respiratory chain Complex I and Complex V subunits, and down regulation of PRA1 domain family member 2 (PRAF2) and ATP synthase inhibitory factor subunit 1 (ATP5IF1), correlates with impaired mitochondrial quality control, potentially leading to the accumulation of damaged, ROS-leaking mitochondria that contributes to cellular pain signaling. The concurrent increase in glutathione reductase (GSR), glutathione S-transferase pi 1 (GSTP1), glutathione S-transferase mu 2 (GSTM2), and glutathione synthetase (GSS) (Supplementary Tables S1 and S7), shows the platelet’s attempt to maintain redox balance against the oxidative stress that trigger crises [70,71,72,73,74,75] In summary, the damaged mitochondria are unable to undergo mitophagy and are incapable of destroying reactive oxygen species. The resulting inflammatory response is directly associated with the pattern and nature of pain in individuals with SCD.

The observed changes in IL-12, IL-17, IL-18 and IL-21 are therefore more appropriately interpreted as an inflammation- and cellular stress–related proteomic signature, rather than an inflammatory syndrome defined solely by CASP3 or HSP90AB4P. The absence of significant changes in most detected interleukins does not exclude altered inflammatory regulation, because these processes may involve apoptosis, cellular stress, autophagy, and downstream cytokine-regulatory pathways.

Protein classifications used in this study represent overlapping pathway associations and should not be interpreted as tissue-specific or mutually exclusive. CASP3 participates in apoptosis and cellular stress and has documented roles in both central nervous system and inflammatory processes. Similarly, ATG7 is a ubiquitously expressed autophagy-related protein that may contribute to inflammation and pyroptosis through regulation of cellular recycling and stress responses. We therefore interpret these proteins within multiple relevant biological pathways rather than assigning them exclusively to a single functional category.

High-pain patients had an enhanced proteomic signature of ubiquilin 2 (UBQLN2) that clustered with proteins usually associated with neurodegeneration including valosin-containing protein (VCP), a protein that shuttles ubiquitinated protein substrates for proteasomal degradation [72] Both proteins play critical roles in cellular quality control and protein clearance. Mutations in these genes are notably linked to neurodegenerative diseases like Amyotrophic Lateral Sclerosis (ALS) and Frontotemporal Dementia (FTD). These findings suggest that chronic pain in SCD is not just peripheral but may involve central sensitization where the nervous system becomes permanently hypersensitive due to repeated neurological injury due to vaso-occlusion and ischemia reperfusion with resulting inflammation [17].

### 4.1. Implications for Pain Mechanisms in SCD

Collectively, these findings suggest that chronic pain in SCD, particularly in individuals with high pain burden, is driven by systemic cellular stress rather than isolated acute nociceptive events. The convergence of mitochondrial dysfunction, impaired mitophagy, oxidative stress, and disrupted proteostasis provides a mechanistic framework linking bioenergetic failure to persistent pain signaling. Defective mitochondrial clearance and sustained reactive oxygen species (ROS) production can promote neuroinflammation and redox-sensitive signaling pathways known to sensitize nociceptive circuits and facilitate the transition from episodic to chronic, neuropathic pain [34,75].

In parallel, metabolic reprogramming marked by suppression of oxidative phosphorylation and increased glycolytic enzyme expression may exacerbate oxidative stress and inflammatory signaling, reinforcing pain susceptibility. Platelet hyper-reactivity and pro-thrombotic signatures further suggest a feed-forward loop linking vascular dysfunction, chronic inflammation, and pain. Together, these data support a model in which chronic pain in SCD reflects integrated dysfunction across mitochondrial, metabolic, and neuroinflammatory pathways, extending beyond traditional nociceptive mechanisms.

Together, these findings have several important implications for understanding pain in SCD. First, they support a role for platelets as accessible peripheral indicators of mitochondrial dysfunction, neuroinflammatory stress, and neuronal sensitization relevant to pain phenotypes in SCD. Second, the identified protein signatures provide a foundation for future studies aimed at validating platelet-derived markers of neuropathic pain and related central nervous system vulnerability. These results highlight potential mechanistic links between systemic inflammation, mitochondrial impairment, and neuroimmune crosstalk across peripheral and central compartments, offering new avenues for investigating how chronic cellular stress contributes to persistent pain in this population. Finally, neurodegenerative-like cellular stress pathways may represent new therapeutic targets for pain in SCD.

### 4.2. Limitations

This study is a proof of principle and feasibility study. The cross-sectional design precludes causal inference regarding the relationships between proteomic alterations and pain phenotypes. In addition, the modest sample size may limit the generalizability of these findings across the broader SCD population. Although the proteomic signatures were robust and consistent across analytical pipelines, functional validation of the identified pathways was beyond the scope of this proof of concept, feasibility study. Future investigations should incorporate longitudinal designs, targeted functional assays, and complementary clinical measures, including neurocognitive assessments and neuroimaging, to directly link platelet-derived proteomic signatures with neurological outcomes and pain trajectories in larger SCD cohorts including comparisons with healthy adults without pain and other pain conditions.

## 5. Conclusions

This proof of concept, feasibility study identifies unexpected and biologically meaningful platelet proteomic signatures in individuals with SCD that are strongly associated with pain phenotypes. Rather than the expected enrichment of canonical nociceptive pathways, the findings implicate coordinated disruptions in mitochondrial function, neurodegenerative signaling, proteostasis, and redox balance. These results underscore the systemic nature of SCD and suggest that chronic pain in this population is driven by interconnected cellular stress responses that extend beyond acute vaso-occlusive events.

This work highlights the potential of platelet proteomics as a powerful and accessible approach for uncovering subclinical pathophysiology in complex diseases. The identification of pathways linked to neurodegeneration and mitochondrial dysfunction supports a model in which persistent bioenergetic failure and oxidative stress contribute to central sensitization and chronic pain in SCD. Together, these findings point to mitochondrial and redox pathways as promising targets for biomarker development and therapeutic intervention, providing a foundation for future studies aimed at improving pain stratification and management in individuals with SCD.

## Figures and Tables

**Table 1:** Inclusion/Exclusion criteria for study participants.

Inclusion	Exclusion
**≥18 years old**	Cognitive or physical impairments that precluded study participation
**African Descent**	Clinical diagnosis of diabetes mellitus or polyneuropathy
**Confirmed diagnosis of SCD**	Legal blindness
**Fluent English**	
**History of moderate to severe SCD-related pain, defined as a self-reported score of ≥3 on a 0–10 numeric rating scale within the preceding 12 months**	
**Chronic pain occurring on more than 50% of days over the prior three months**	

**Table 2:** Description statistics of participants according to pain.

	High (N = 8)	Low (N = 8)	*p*-Value
Gender			
Female	2 (25.0%)	6 (75.0%)	0.126
Male	6 (75.0%)	2 (25.0%)	
Age (years)			
Mean (SD)	42.4 (6.46)	42.9 (9.83)	0.906
Median [Min, Max]	43.0 [33.0, 50.0]	46.5 [29.0, 54.0]	
SC.Genotype (%)			
SC	2 (25.0)	1 (12.5)	1
SS	6 (75.0)	6 (75.0)	
S Beta + thal	0 (0)	1 (12.5)	

## Data Availability

The original contributions presented in this study are included in the article/Supplementary Materials. Further inquiries can be directed to the corresponding author.

## Supplementary Materials

The following supporting information can be downloaded at: https://www.mdpi.com/article/10.3390/medsci14050517/s1, The supplementary materials include Excel spreadsheets containing proteomic data and pathway enrichment analyses that support the findings reported in the manuscript. Table S1 provides the complete proteomic data table. Tables S2–S11 present associated functional annotation and enrichment results, including respiratory electron transport and ATP synthesis, biological processes, cellular components, molecular functions, KEGG pathways, Reactome pathways, disease-related annotations, and mitophagy-related proteins. Table S1: Proteomic Table; Table S2: FDR and Fold Change; Table S3: Respiratory electron transport ATP synthesis; Table S4: Biological Processes; Table S5: Cell Component; Table S6: Molecular Function; Table S7: KEGG; Table S8: Reactome; Table S9: Diseases; Table S10: Mitophagy; Table S11: Cytokine_Proteins.
